# Virulent *Drexlervirial* Bacteriophage MSK, Morphological and Genome Resemblance With Rtp Bacteriophage Inhibits the Multidrug-Resistant Bacteria

**DOI:** 10.3389/fmicb.2021.706700

**Published:** 2021-08-24

**Authors:** Muhammad Saleem Iqbal Khan, Xiangzheng Gao, Keying Liang, Shengsheng Mei, Jinbiao Zhan

**Affiliations:** Department of Biochemistry, Cancer Institute of the Second Affiliated Hospital (Key Laboratory of Cancer Prevention and Intervention, China National Ministry of Education), School of Medicine, Zhejiang University, Hangzhou, China

**Keywords:** electron microscopy, rosette like tail, *Rtp*_*like* bacteriophage, *Drexlerviridae*, antibacterial activity

## Abstract

Phage-host interactions are likely to have the most critical aspect of phage biology. Phages are the most abundant and ubiquitous infectious acellular entities in the biosphere, where their presence remains elusive. Here, the novel *Escherichia coli* lytic bacteriophage, named MSK, was isolated from the lysed culture of *E. coli* C (phix174 host). The genome of phage MSK was sequenced, comprising 45,053 bp with 44.8% G + C composition. In total, 73 open reading frames (ORFs) were predicted, out of which 24 showed a close homology with known functional proteins, including one tRNA-arg; however, the other 49 proteins with no proven function in the genome database were called hypothetical. Electron Microscopy and genome characterization have revealed that MSK phage has a rosette-like tail tip. There were, in total, 46 ORFs which were homologous to the Rtp genome. Among these ORFs, the tail fiber protein with a locus tag of MSK_000019 was homologous to Rtp 43 protein, which determines the host specificity. The other protein, MSK_000046, encodes lipoprotein (cor gene); that protein resembles Rtp 45, responsible for preventing adsorption during cell lysis. Thirteen MSK structural proteins were identified by SDS-PAGE analysis. Out of these, 12 were vital structural proteins, and one was a hypothetical protein. Among these, the protein terminase large (MSK_000072) subunit, which may be involved in DNA packaging and proposed packaging strategy of MSK bacteriophage genome, takes place through headful packaging using the pac-sites. Biosafety assessment of highly stable phage MSK genome analysis has revealed that the phage did not possess virulence genes, which indicates proper phage therapy. MSK phage potentially could be used to inhibit the multidrug-resistant bacteria, including AMP, TCN, and Colistin. Further, a comparative genome and lifestyle study of MSK phage confirmed the highest similarity level (87.18% ANI). These findings suggest it to be a new lytic isolated phage species. Finally, Blast and phylogenetic analysis of the large terminase subunit and tail fiber protein put it in Rtp viruses’ genus of family *Drexlerviridae.*

## Introduction

Bacteriophages are the most abundant acellular biological entities, with an estimated 10^31^ phage particles in the biosphere (Keen, 2015). They are obligate intracellular parasites, present everywhere wherever bacteria live (Suttle, 2005). The roles of phages could not be ignored as they are used as vehicles in gene transfer (Gómez-Gómez et al., 2019), an alternative to antibiotics (Alvi et al., 2020), and antibody engineering using phage display technology (Kalim et al., 2019). Regarding benefits, bacteriophages also cause severe problems in industrial production and fermentation units. Previously identified bacteriophage Rtp and DTL were isolated from the lysed culture in the industrial firm. Furthermore, a recent study conducted by Pacífico et al. (2019) isolated 43 coliphages from the human body fluid specimens, including blood, urine, and tracheal aspirates. Maintaining a sterile laboratory and working environment exacerbates the problem because these entities are often less vulnerable to standard sterilization (Wietzorrek et al., 2006; Halter and Zahn, 2018; Sprotte et al., 2019).

*Escherichia coli* C, a significant Gram-negative bacterium primarily isolated from sour cow’s milk by Ferdinand Hueppe in Germany. This *E. coli* strain was commonly used in the industrial fermentation process like propanol, butanol, and 3-hydroxypropanoate under anaerobic conditions (Monk et al., 2016; Pekar et al., 2018). To our best knowledge, only one temperate phage P2 of *E. coli* C has been reported by Bertani and coworkers to date (Wiman et al., 1970). The lytic single-stranded bacteriophage Phi X 174 was also isolated from *E. coli* C, having the smallest size and no tail. Phage X 174 is the first bacteriophage with the whole genome, sequenced by Sanger, belonging to the *Microviridae* family (Sanger et al., 1977; Zheng et al., 2009).

However, the number of the bacteriophage genomic world is incomplete and lags far behind their host bacteria (Clokie et al., 2011). Horizontal gene transfer between phage–host interactions contributes to the genetic diversity and evolution of the phage community (Chibani-Chennoufi et al., 2004). The emergence of antibiotic resistance against bacteria is a significant threat to public health due to the lack of new antimicrobial compound discovery. Colistin is a last-alternative defense against carbapenem-resistant bacterial infections (MCR1/4). So, a new executing strategy needs to be adopted. In this regard, phage therapy has opened new avenues for treating multidrug-resistant (MDR) and extensively drug-resistance (XDR) bacterial pathogens. For safety reasons, biosafety assessment of the phage should be done before use, as phages may act as a vehicle to carry the antibiotic resistance and virulence genes (Cui et al., 2017). A previous study was done by Granobles Velandia et al. (2012), who isolated a phage in which the genome contained Shiga toxins (*Stxs*). These findings suggested that genome sequencing is necessary for safety concerns.

Consequently, with the continuous development of Next Generation Sequencing (NGS) techniques, more than 8,000 phages have completed genome-wide sequencing (Salisbury and Tsourkas, 2019). Then classifying the bacteria based on shape, nucleic acid, size, and lifestyle as either temperate or lytic phages got attention; they played a crucial role in the horizontal gene transfer and combating antibiotic resistance in medicine (Ackermann, 2011; Cui et al., 2017). It is, therefore, immensely desired to explore and scratch the surface to determine the sequences of coliphages because phage genomics provides the evidence to trace phage and bacterial evolution.

Therefore, in this study, we report the isolation of a lytic phage of *E. coli* C named MSK bacteriophage from the lysed culture. MSK bacteriophage can cause rapid lytic infection of bacterial culture in less than 2 h. Numerous bacteriophages in modern industrial fermentation facilities or labs can be a severe problem, including quality and quantity of products and financial losses (Halter and Zahn, 2018). To better understand the lifestyle events, genome, critical investigations, and its possible therapeutic usage, we sequenced the genome of MSK bacteriophage.

This study is the first report of isolation, identification, and characterization of the virulent phage from *E. coli* C having double-stranded DNA and a rosette-like tail. Similar previous illustrations revealed that over 96% of phages are tailed and contain dsDNA (Ackermann, 2011). Furthermore, ViPTree, virfam, and comparative analysis showed that the phage MSK is a member of the *Drexlerviridae* family (*Siphoviridae*), *Braunvirinae* subfamily, and Rtp like viruses genus in terms of the genomic content capsid, tail structure, and protein composition. In addition, MSK phage possesses a tail fiber protein together with a series of 46 other ORF showing the recognizable homology with Rtp bacteriophage (T1 like bacteriophage) and also has morphological resemblance to the rosette-like tail morphology. Indeed, phage MSK was shown to have a narrow host range, infecting the *E. coli* strains (AMP, TCN, and Colistin), and is highly stable even at high temperatures (100°C) and pH. Overall, these studies in the future could provide detailed insights regarding phage therapeutic drugs, phage virulence, antibiotic gene finding, and tracing the phage evolution and diversity.

## Materials and Methods

### Bacteriophage Enumeration and Purification

*E. coli* phage MSK was primarily isolated from lysed *E. coli* (Migula) Castellani and Chalmers ATCC^®^13706^TM^, also called “*E. coli* C” which is the primary host of bacteriophage ΦX174 (Fujimura and Kaesberg, 1962). Phage MSK was subsequently propagated on *E. coli* C in Luria Bertani broth (0.5% salt). In detail, 250 mL of an *E. coli* culture was grown at 30°C until optical density (OD) 600 nm reached 0.4. Then phage suspension was inoculated for 4 h at 30°C at MOI 0.01 until the culture OD600 to 0.4. After the cell lysis, culture debris was pelleted by centrifugation at 7,155×g for 15 min at 4°C. The crude phage suspension was concentrated at 4°C with one-fifth of the lysate volume of 20% polyethylene glycol (PEG) 8,000 and 1.5 M NaCl for 4 h. Next, phage suspension with PEG was centrifuged (16,099 × g for 15 min at 4°C), and the phage pellet was suspended in 8 mL of phosphate buffer (pH = 7.4). Finally, phage was collected by centrifugation at 16,099 × g for 15 min, and residual cell debris was removed.

Phage concentration was done in the presence of a 12% sucrose bed by using ultracentrifugation (105,000 × g for 2 h at 4°C), and then the phage pellet was kept in PBS 300 μL overnight at 4°C. Further purification of phage particles was performed using discontinuous sucrose and iodixanol gradient ultracentrifugation (175,000 × g for 4 h at 4°C) which have been widely used to separate virus particles and vectors on a large scale (Zolotukhin et al., 1999). The purified band was separated and gradient media was removed using Amicon^®^ Ultra-4 Centrifugal Filter Unit (100 kDa). The purified phage titer was determined by plaque-forming unit (*PFU/mL*), as already described (Clokie et al., 2009). This phage suspension (1.5 × 10^10^ *PFU*/mL) was stored at 4°C and used for subsequent experiments. Overall systematic representation of MSK bacteriophage enumeration and purification is shown (Supplementary Figure 1).

### Electron Microscopy

A purified bacteriophage MSK sample was examined by transmission electron microscopy (TECNAI-Spirit 120 Kv) in The Center of Electron Microscopy, Zhejiang University School of medicine. Briefly, the carbon-supported grid was glow discharged using the PLECO EASI GLOW^TM^ 91000 Glow discharge system. Then 10 times diluted sample (3 μL) was absorbed to the glow discharged grid for 30 s, and extra phage particles were swiped out with filter paper. Then the grid containing the phage was stained with 2% aqueous uranyl acetate (UA) with three times intervals of 10 s, 10 s, and 1 min. The grid was dried at room temperature for at least 1 min, and micrographs were taken at different magnification using 87 Kx and 105 Kx with FEI Tecnai G2 Transmission Electron Microscope.

### Assessment of MSK Phage Potential to Reduce Bacterial Growth

A known reduction assay, as reported earlier, was used for bacterial growth retardation (Clokie et al., 2009). For this purpose, a known amount of bacterial culture of *E. coli* C (*CFU*/mL) was treated with phage MSK in three independent reactions at MOI 10, 1, and 0.1 incubated at 30°C with shaking for 4 h. One flask was considered as a control (without phage MSK) and incubated at 200 rpm. Growth was estimated by measuring the optical density (OD600) every 30 min for 4 h.

### MSK Phage Stability Under Physiochemical Conditions

The different physiochemical conditions (pH, temperature, and UV light) affect the stability of bacteriophage, which directly affects the titer or MOI (Multiplicity of infection) of phage. For this purpose, the known phage titer (1.5 × 10^10^ *PFU*/mL) was treated at different pH values (4, 6, 7.4, 8, 10, and 12) and different temperatures (30, 37, 45, 60, 100, and 120°C) for 1 h and UV light (20 W) for an additional period (5, 10, 15, 25, and 60 min). Finally, the viral titer was determined by the double-layer plaque assay method.

### Determination of Burst Size and Burst Time for MSK Bacteriophage

It is imperative for the bacteriophage analysis to measure the one-step growth curve, which helps us calculate the latent period and burst size. Briefly, the latent time was calculated from the single-stage growth curve. The burst size was calculated by the mean yield of phage used for a bacterial infection to the mean output of phage particles liberated after infection. This *in vitro* experiment was performed according to the method reported previously (Alvi et al., 2020). Briefly, bacterial host strain (1 × 10^10^ *CFU*) was harvested by centrifugation (1,789 ×g) for 5 min. Then, supernatant was discarded, and the pellet was mixed in 500 μL of LB broth. Further, bacterial culture was mixed with MSK (1 × 10^10^ *PFU/mL*) at MOI 1, followed by incubation for 1 min at 30°C. After that, free bacteriophage was removed from the mixture after centrifugation at 13,000 rpm for 30 s, and the titer in the supernatant was measured. Next, the pellet was dissolved in 100 mL of fresh sterile LB broth, followed by incubation at 30°C. After every 5 min interval, samples (1 mL) were collected for 1 h; subsequently, the phage titer was calculated by plating on an LB agar plate.

### Genomic DNA Characterization and Library Generation

The genomic DNA of purified phage *E. coli* phage MSK was firstly extracted using the M13 DNA extraction kit (Omega) according to the manufacturer’s protocol. Further, DNA was run on 0.8% agarose gel to get rid of other DNA contaminants. Then DNA was purified from a gel extraction kit (Omega). The concentration of the DNA sample was determined by Equalbit^TM^ dsDNA High sensibility assay kit (Vanzyme, China, Cat No; EQ111-01/02) by using Qubit^®^ 2.0 fluorometer and agarose gel electrophoresis to detect the quality of the DNA sample. Next, DNA was treated with *DNase I* and *RNase I* at 37°C overnight to make sure either DNA has single-stranded or double-stranded as previously described (Ma and Lu, 2008).

Initially, the size (base pair) of the genome was assessed through digestion with molecular scissors before sequencing. The phage MSK DNA was digested with a single and combination of enzymes. The single Fast-Digest enzymes included *EcoRI, NheI, XbaI, SacI, NdeI, BglII*, and *XhoI.* Similarly, the double digestion enzyme included these combinations: *XbaI + PacI and BamHI + PacI, PacI + SalI*. All enzymes were purchased from New England BioLabs^®^ Inc. All the digestion were performed at 37°C with overnight incubation. The restriction fragments were mapped on 0.8% agarose gel and stained with red staining dye (Szymczak et al., 2020). For further characterization of DNA, electropherogram of *EcoRI* digested DNA was mapped with Agilent Fragment Analyzer Systems (Hangzhou Lianchuan Biotechnology Co., Ltd., China) to estimate the genome size (Lakha et al., 2016; Yu et al., 2016).

The genomic DNA of phage MSK was subjected to next-generation sequencing platforms (MGISEQ-2000 Beijing Genomics Institute) at the State Key Laboratory of agriculture science Zhejiang University, China. The DNA samples that have passed the electrophoresis detection were randomly broken into pieces with a length of approximately 350 bp using a Covaris ultrasonic disruptor. Then the processed DNA fragments were repaired using an A-tail sequencing adapter at the end. After that constructed library was quantified using Agilent 2100 and also ensured the quality of the library. Finally, different libraries were pooled for sequencing.

### Sequence Assembly and Genome Analysis

Raw data obtained from sequencing were cleaned to make it accurate and reliable for subsequent analysis. Before sequence assembly, 15 K-mer statistics were performed to estimate the size of the gnome. Sequence reads were assembled through SOAP denovo (version 2.04), SPAdes, ABySS, and finally use CISA software for integration. Gapclose (Version: 1.12) was used to fill the gap, Scaffold length greater than 500 bp was selected for further evaluation, and statistical analysis was performed. The genome annotation was performed utilizing different bioinformatics tools. Online RAST server, Gene MarkS (Version 4.17), and Gene Glimmer were used for the identification of open reading frames (ORFs) within the genome.

In addition, the Genome sequence was analyzed using RepeatMasker (Version open-4.0.5) software for scattered repetitive sequence prediction and Tandem Repeats Finder (Version 4.07b) for tandem repeats in DNA sequences. Furthermore, the genome was scanned by tRNAscan-SE software (Version 1.3.1) for tRNA prediction, rRNA was predicted by scrutinizing through rRNA library and rRNAmmer software (Version 1.2), and cmsearch program (Version 1.1rc4) was used to determine the final sRNA. The genome was also analyzed for gene island (genomics island, GIs) to ensure the transfer of horizontal gene transfer, which is predicted by comparative genome analysis and CRISPR-associated genes (Cas gene) in the genome was identified by using the CRISPR finder tool.^1^ Finally, the sequence was analyzed using phiSpy software (Version 2.3) to predict prophage in the genome sequence.

Genome function annotation was done by comparing the ORFs with already reported genes using GO, KEGG, COG, NR, Pfam, TCDB, and swiss-port. Gene ontology (GO) was used to predict the cellular components, molecular function, and biological process in the genome. Metabolic pathways in the genome were predicted by KEGG^2^ using multiple tools like KEGG pathway, KEGG drug, KEGG disease, KEGG module, KEGG Genes, KEGG genome, and KEGG orthology. NCBI COG protein database^3^ was used to find the coding protein of the complete genome, which determined their features.

Transporter Classification Database (TCDB) was used to identify the membrane proteins in a sequence. Identifying of protein domains is particularly crucial for analyzing protein function done through the Pfam database.^4^ Swiss-pro^5^ provides a high level of annotation results like function, domain structure, post-translational modification, and variations. The enzyme family search was done by Carbohydrate-Active enZYmes Database (CAZy) was also utilized. Furthermore, comprehensive species information was obtained by using NR (non-redundant) Database. SignalP (Version 4.1) and TMHMM (Version 2.0c) were used to identify sequence elements involved in targeting the secretory pathway and transmembrane helices in annotated gene products, respectively.^6, 7^

The genome sequence was also analyzed in seven different types (T1SS, T2SS, T3SS, T4SS, T5SS, T6SS, T7SS) of TNSS (type N secretion system), which are usually secreted into the extracellular environment and cause pathogenic response and cell death. For T3SS effector protein prediction, *EffectiveT3* software (Version 1.0.1) was used. The secondary metabolites play a role during specific growth periods, but these proteins have no apparent function. So, secondary metabolites prediction was made by using the antiSMASH program (version 2.0.2). Pathogen and host interaction database (PHI) was used to find the target genes responsible for the host infection.

The function of predicted ORFs and their similarity to other phage genes was analyzed using NCBI BLASTp,^8^ HMMER,^9^ and MPI Bioinformatics Toolkit, HHpred.^10^ In addition the proteins were characterized for their theoretical molecular weight and isoelectric point (pI) using ProtParam (ExPASy—ProtParam tool). Finally, the genome map of the predicted ORFs and their GC percentage in the whole genome was drawn with the help of Snapgene.^11^

### Genome Sequence Accession Number

The complete genome sequence of the *E. coli* phage MSK has been submitted in the NCBI GenBank (Bankit) nucleotide sequence database with accession number MW057918.

### Safety Assessment of the Phage MSK

Lifestyle (lytic or temperate) of the *E. coli* phage MSK was predicted using the PHACTS program. Then all the interpreted ORFs were compared against the sequences in the comprehensive antibiotic resistance database (CARD),^12^ which analyzed the sequences using BLAST and resistance gene identifier (RGI) software for prediction of resistome based on SNP and homology model. RGI uses a perfect, strict, and complete algorithm to identify the antimicrobial resistance (AMR) genes in the entire genome (Alcock et al., 2019). In addition to AMR genes determination, we also determine virulence factor by virulence Finder database,^13^ for detection of *E. coli* virulence genes [verocytotoxin 1 (vtx1), verocytotoxin 2 (vtx2), and intimin (eae)] and subtypes (Joensen et al., 2014).

### SDS PAGE and *in silico* Mass Fingerprinting

Phage MSK structural Protein profile was determined by Sodium Dodecyl Sulfate-Polyacrylamide Gel Electrophoresis (SDS-PAGE). The purified phage sample (20 μL) having phage particles was dissolved in 20 μL laemmli buffer, and the mixture was heated in boiling water (90°C) for 10 min. After heating, a medley of the sample was subjected to post cast, horizontal 12% SDS-PAGE gel electrophoresis with protein marker (pre-stained protein ladder, Thermo Fisher Scientific) and Tris-glycine as running buffer. The gel was stained with Coomassie Brilliant Blue R250 dye (Sigma), destained with the destaining solution for 30 min, and visualized under the camera. Peptide mass fingerprinting was performed *in silico* using Gel Molecular weight analyzer (Origin(Pro), 2021). The software calculated the peptide mass (kDa) by known molecular weight protein, a standard curve generated by a polynomial with order three and regression equation (*R* = 0.99927). Finally, the calculated molecular weight was compared against a feature table of MSK phage proteins manually, and then protein residues and molecular weight was calculated using Protein Molecular Weight (bioinformatics.org) and domains.

### DNA Packaging Strategy and Comparative Genome Analysis

The identification of genome termini and prediction of the phage packaging mechanism was carried out using the PhageTerm application incorporated in Galaxy^14^ (Garneau et al., 2017). The Classification of phage MSK major proteins (neck, head, and tail protein) was constructed using Virfam online automated tool, enabling us to predict the phage families. For this purpose, the annotated protein sequence of phage MSK was run online to predict the structural module (head, neck, and tail genes) and their function. Virfam can also predict the morphologic type and classify the phages based on the “head, neck, and tail” module. Primarily these analyses can be used to classify unknown viruses (Lopes et al., 2014).

Furthermore, the tree among *E. coli* phages MSK and other bacteriophages of different families were generated by ViPTree server version 1.9.^15^ ViPTree (proteomics tree) was generated based on the genome wide sequence similarities computed by tBLASTx. The proteomics tree method is productive to scrutinize the genomes of the newly sequenced bacteriophages or viruses for the prediction of families.

Additionally, comparative genome analysis calculates the average nucleotide identity (ANI) using the ANI calculator (Yoon et al., 2017). Finally, the genome of MSK phage was compared with the *Drexlerviridae* family’s four bacteriophages having (*Escherichia* phage vB_EcoS-2862V, *Escherichia* phage vB_Ecos_CEB_EC3a, *Escherichia* phage DTL and, Rtp bacteriophage) having ANI value greater than 83%. These bacteriophages have similar lengths (44–46 Kbp), the number of genes (67–75). The genome of the Rtp bacteriophage was downloaded in the in gene bank file (gb) as a reference genome, and the other three phage genome was downloaded in FASTA format. Comparative genomic analysis was done using multiple sequence alignment using MAUVE (Darling et al., 2004).

### Antibacterial Activity of MSK Phage Against Multidrug-Resistant Bacteria

Antimicrobial activity of bacteriophage MSK was assessed on five clinical antibiotic resistance *E. coli* isolates acquired from the first affiliated hospital and Department of Pathogen Biology and Microbiology, Zhejiang University School of Medicine, China. These *E. coli* isolates were resistant to different antibiotics like ampicillin (AMP), Extended Spectrum Beta-Lactamase (ESBL), Tetracycline (TCN), Colistin (mcr-4), and Colistin (nmcr-1). The lytic activity of MSK was determined against these five *E. coli* pathogenic strains, *Pseudomonas syringae* and *Salmonella anatum*, using a spot test. Briefly, 100 μL of a log-phase culture of the test strain was mixed gently by avoiding bubble formation with 3 mL of LB semi-solid agar. The mixture was poured on the solidified LB agar plate and spread over the plate uniformly by swirling. Then 3 μL of purified, diluted phage (1.35 × 10^5^ *PFU/mL*) was added to the solidified LB agar plate having different pathogenic strains. The appearance of clear zones after overnight incubation at 37°C was presumptive of positive lytic activity of MSK (Clokie et al., 2009).

## Results

### MSK Bacteriophage Form Clear and Transparent Circular Plaque

**FIGURE 1:**
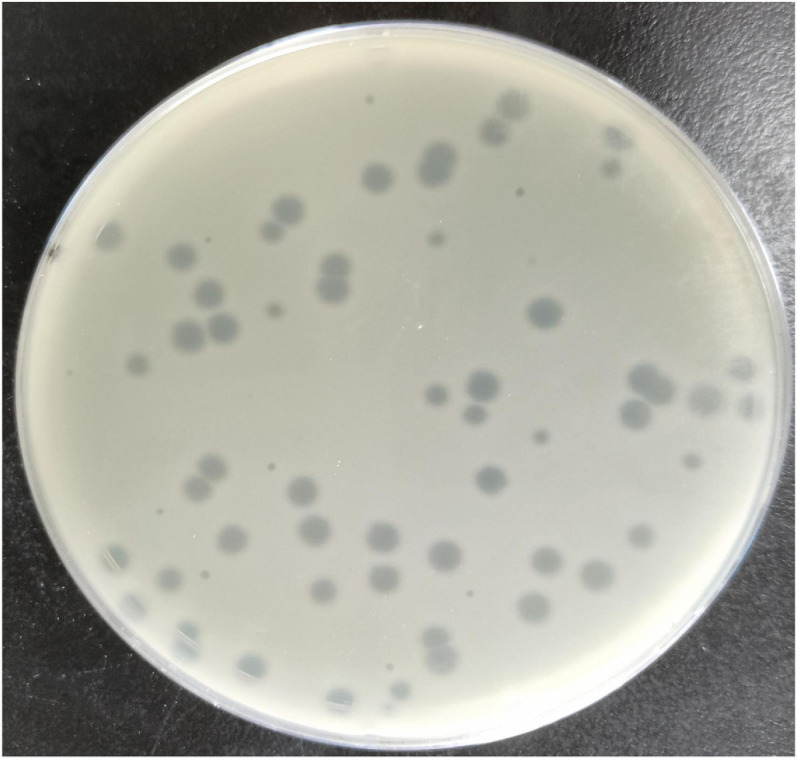
Clear plaque fashioned by *Escherichia coli* phage MSK with *Escherichia coli C* host (primarily ΦX174 host) lawn on a double-layer agar plate.

The *Escherichia* phage MSK indicated a potent lytic activity against *E. coli* C. This phage, named MSK in recognition of its identifier, lysed the *E. coli* culture in the Gene and Antibody Engineering lab. The lysed culture was diluted and cultured to obtain transparent plaques on a double-layered plaque assay. Phage MSK produced circular and homogenous transparent plaques with a diameter of around 4–5 mm (Figure 1). The titer of the phage MSK was against host strain 10^8^ *PFU*/mL. Finally, three times purified, a clear plaque was picked and propagated; lysate obtained was preserved at 4°C for further studies.

### Characterization of Phage Morphology Through Electron Microscopy

**FIGURE 2:**
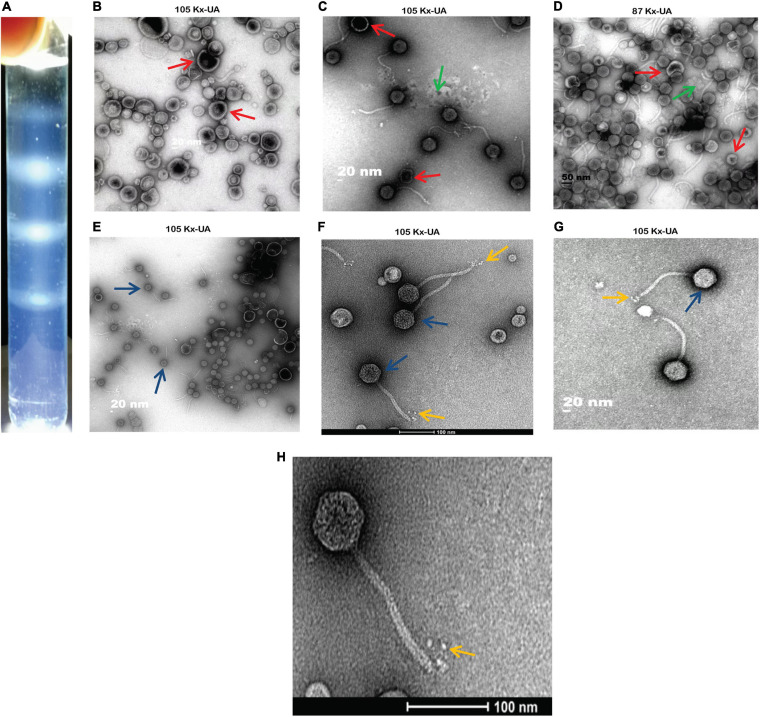
A comparison between two density gradient media and Electron Micrograph of Escherichia phage MSK **(A)** cleared white band was observed in iodixanol gradient compared with sucrose gradient. **(B–D)** Three different bands separated during discontinuous sucrose density gradient. **(E–G)** Three various bands separated during discontinuous iodixanol density gradient. Red arrow: broken particles, blue arrow: intact particles, yellow arrow: rosette-like tail, green arrow: opaque background. **(H)** Electron micrograph of Escherichia phage MSK having a rosette-like tail with 100 nm bar line.

Using the discontinuous sucrose and iodixanol gradient, three different negative staining results from each gradient media were obtained. The white strips corresponding to the location of particles were taken out from the gradient, and further negative staining of electron microscopy was performed for morphological characterization. From the results, the discontinuous sucrose gradient MSK particles were found to be broken non-infectious with opaque background having partial morphology. Most of the phage particles separated by this gradient always lost their DNA, tail and retained only capsid protein. Three different bands were separated during the discontinuous sucrose gradient. The first band micrograph represents the broken head from the tail and empty capsid with an opaque background (Figure 2B). The second micrograph depicts an intact particle with opaque background (Figure 2C). The third micrograph represents the particles which deformed and collapsed because of the centrifugation speed and gradient media (Figure 2D). Compared to sucrose gradient media, the iodixanol gave many essentially complete intact MSK phage particles and transparent background. The first micrograph represents some phage particles with empty shell, but many were intact compared to sucrose (Figure 2E). The second and third iodixanol EM micrograph found it to be a complete infectious particle containing DNA, capsid, and tail with transparent background (Figures 2F,G). The second and third bands obtained from iodixanol gradient with complete infectious and morphological features were used to further structure characterization.

Consequently, morphological characterization of phage MSK revealed a non-enveloped, long, non-contractile tail of approximately 151 nm in length and 8 nm in width, with four short linked rosette-like morphologies of the tail tip through electron micrographs. Moreover, it has an icosahedral head with an average diameter of 50 nm. Thus, MSK phage has a close morphological resemblance with Rtp bacteriophage based on the icosahedral head, tail morphology, and tail length.

### MSK Lyse the Host Bacteria Growth for 4 h at Different MOI

**FIGURE 3:**
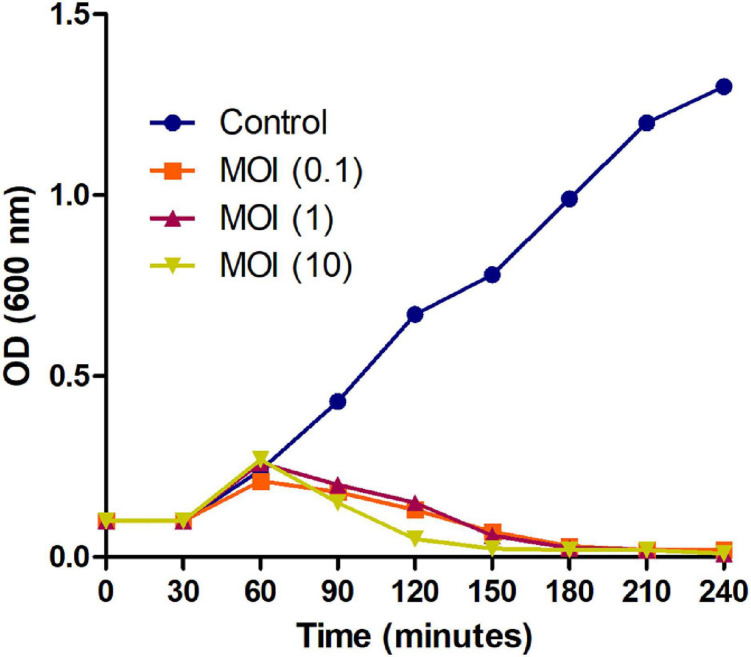
Bacterial growth reduction assay of MSK bacteriophage at different MOI. Purified lytic bacteriophage *in vitro E. coli* C was infected with different phage MOIs (10, 1, and 0.1). *E. coli* C growth reduction assay of MSK at MOI = 10 showing a marked decrease in the bacterial growth within 2 hours as compared to MOI (0.1, 1) and non-treated *E. coli* C control. The bacterial growth was utterly suppressed till 3–4 hours, infecting with the MOI = 0.1,1,10.

Further, to check the lysing activity of the Phage MSK against host bacteria, we applied three MOIs (10, 1, and 0.1) along with control. Phage MSK completely inhibits the host bacterium growth *in vitro* for 4 h when different phage titers were applied in contrast to control. The differences between these groups become insignificant (Figure 3). The optical density of the phage inoculated group was much lower than that of a control group which indicated that MSK phage could completely lyse the host cell within 2 h of infection. After 2 h, the number of host cell OD becomes close to zero in growing media.

### MSK Stability Under Different Physiochemical Conditions

Evaluation of the phage stability and its activity in different physiochemical conditions (pH, temperature, UV light) plays a significant role. Results in the pH range of 4–12 showed that phage MSK was less susceptible to all pH ranges, demonstrating MSK phage stability over a wide range. However, we were unable to identify the optimum pH range for MSK phage stability (Supplementary Figure 2A). Similarly, phage stability was evaluated at different temperatures ranging from (30°C to autoclave temperature. The plaque forming unit (PFU) showed that the phage was stable even at high temperatures (100°C). There was not a significant difference noted after applying different temperatures up to 100°C. The phage MSK did not make any plaques at a higher temperature of 120°C. One thing that was interesting to note was that phage MSK makes plaque with a smaller size at a higher temperature of 100°C. That shows that the phage was stable at 100°C (Supplementary Figure 2B).

Additionally, The UV light exposure for MSK phage stability also did not affect its stability. These findings demonstrated that phage is very stable even during the application of harsh conditions (Supplementary Figure 2C). In the last, we were unable to identify the reason behind this stability of the phage.

### MSK Has a Short Latent Period and Burst Size

**FIGURE 4:**
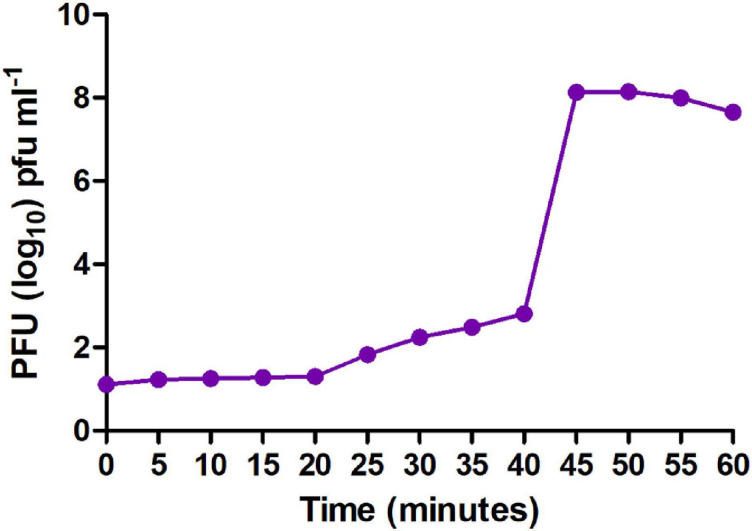
One-step growth curve of MSK bacteriophage. The purified bacteriophage showed a latent period of 25 min, and burst time occurred between 25 and 45 min.

Moreover, to enumerate the phage latent period and burst size, we determined a one-step growth curve. The one-step growth curve showed that the phage MSK had a latent period of 25 min which produces more than 200 virions per cell. The burst time occurred between 25 and 45 min (Figure 4). Thus, the number of phage particles in the supernatant media increased rapidly, specifying that bacteria were lysed and phages were released into the environment.

### MSK Phage DNA Quantification

**FIGURE 5:**
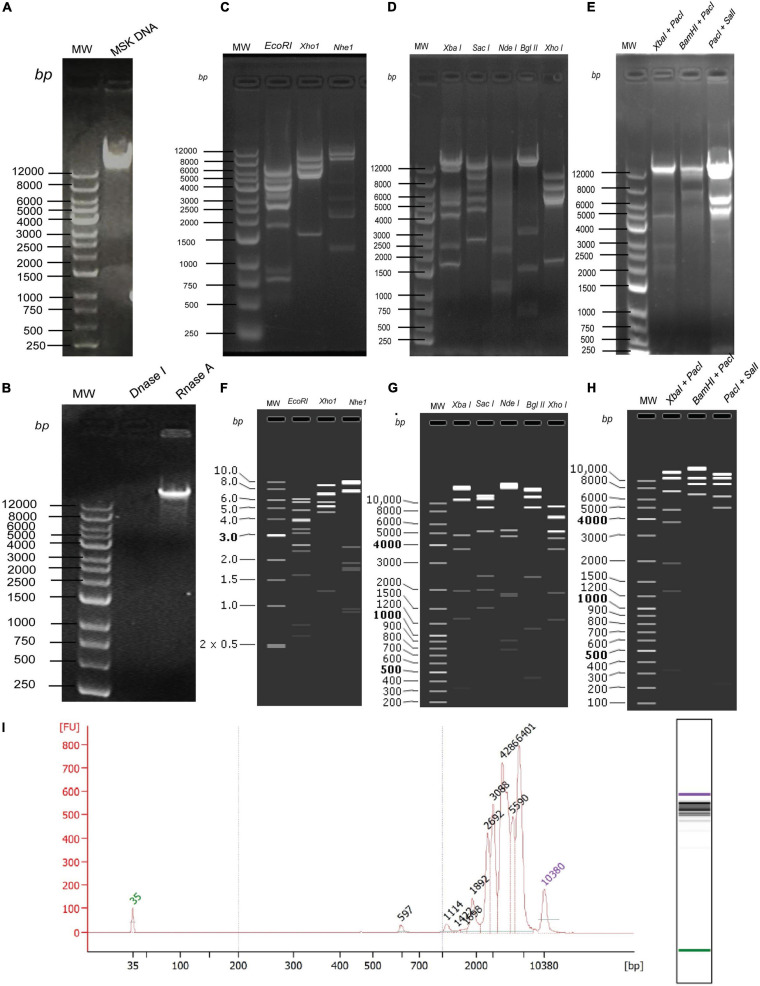
*In vitro* and *in silico* phage MSK size estimation through restriction enzymes. **(A)** Purified DNA extracted from purified phage particles. **(B)** Phage genome confirmation using *DNase* I and *RNase* I. **(C)** Phage DNA digested with single enzymes digestion *EcoR*I, *Xho*I, and *Nhe*I. **(D)** Phage DNA digested with single enzymes digestion *Xba*I, *Sac*I, *Nde*I, *Bgl*II, *Xho*I. **(E)** Phage DNA digested with double enzymes digestion *Xba*I + *Pac*I and *BamH*I + *Pac*I, *Pac*I + *Sal*I. **(F)** *In silico* Phage DNA digested with single enzymes digestion *EcoR*I, *Xho*I, and *Nhe*I. **(G)** *In silico* Phage DNA digested with single enzymes digestion *X*baI, *Sac*I, *Nde*I, *Bgl*II, *Xho*I. **(H)** *In silico* Phage DNA digested with double enzymes digestion *Xba*I + *Pac*I and *BamH*I + *Pac*I, *Pac*I + *Sal*I. **(I)** Phage DNA digested *EcoR*I and mapped with Agilent Fragment Analyzer Systems.

Next, to determine the size of phage MSK, the DNA was purified (16.5 ng/μL) from the iodixanol purified bacteriophage and run on 0.8% agarose gel (Figure 5A). DNA was treated with *DNase I* and *RNase I* (Figure 5B), which confirmed that the genome is a linear double-stranded molecule. Firstly, before sequencing, the genomic size was estimated through single digestion with molecular scissors (Figures 5C,D), through which size estimation was indistinguishable. Then, DNA was digested with a combination of enzymes (Figure 5E), which also gave ambiguous band separation and size estimation. Further, the size and integrity of genomic DNA were quantified using the Agilent Fragment Analyzer Systems. Previously, single *EcoRI* digested with clear band of separation was brought to mapped electropherogram. Moreover, the appropriate size before preparing libraries for sequencing was also determined (Figure 5I). Finally, single, double enzyme digested, and electropherogram gave the estimation yield a contig of 40–43 kb in phage MSK genomic DNA.

Secondly, we sequenced the genomic DNA of *E. coli* phage MSK comprising 45,053 bp. Then, the DNA sequence was digested with the same enzyme in SanpGene to confirm the band pattern as shown (Figures 5F–H). These restriction digestion patterns *in silico* and *in vivo* assure the correct genome assembly.

### Genome ORF Features Prediction and Module Formation

The assembled genome of *E. coli* phage MSK comprised 45,053 bp with a GC composition of 44.8%. During the sequence, NCBI blast analysis revealed that the phage genome was similar to previously sequenced *E. coli* phages with 88.44–90.36 percentage identities, including *Escherichia* phage vB_Ecos_CEB_EC3a, Enterobacteria phage vB_EcoS_ACG-M12, *Escherichia* phage DTL and Bacteriophage RTP. With the advancement of sequencing technology, a number of sequenced bacteriophages has been increasing continuously in the NCBI database. Assembled genome NCBI blast showed that the sequence was similar to previously sequenced 32 Escherichia phages displaying 90–91.18% identity during NCBI blast including *Escherichia* phage vB_EcoS-2862V (MK907276.1) and *Escherichia* phage vB_EcoS-2862IV (MK907275.1) revealed 91.18% nucleotide percentage identity and *E*-value = 0. These sequence similarity results suggest that the MSK phage is a member of the *Drexlerviridae* family (tail bacteriophage).

Gene annotation predicted a total of 73 open reading frames (ORFs). Gene Marks showed 71 (Supplementary Figures 3A,B), while gene glimmer and RAST sever predicted 73 ORFs, respectively (Supplementary Table 1). Each ORF had an average length of 564–600 bp, having 39.39% internal GC contents. The gene length distribution showed that ORF covers all the genome but is majorly distributed at the start of the genome (Supplementary Figure 3C). The persistence of repeat sequences has also been found in phage genomes like LTR/DNA/LINE, in different numbers like one, one, and two, respectively. Similarly, Tandem repeats (TR) sequence presence was also found in the genome, but analysis showed no single sequence of SINE, RC, minisatellite, and microsatellite (Supplementary Figures 4A,B).

Further, we also performed a tRNAscan program (version 2.0) analysis that showed only one Arg type tRNA of 78 bp in length, but no sRNA or rRNA presence was noticed in the genome (Supplementary Figure 5A). The genome analysis was also done by finding a CRISPR case-associated gene, but not a single sequence was identified. Finally, Phispy predicted that the genome has similarities with bacteriophages with an average length of 21,847 bp (16,781–38,627), which showed that our genome has all major structural and functional proteins for bacteriophage assembly (Supplementary Figure 5B).

Ahead, genome functional profile was predicted majorly by comparing the protein sequence with available databases with *e*-value ≤ 1e-5 and with the highest score value (default identity ≥ 40%) and for annotation coverage percentage should be higher than 40 (coverage ≥ 40%). Genome functional annotation was done using different algorithms. Using Gene Ontology, we predicted a total number of 22 proteins that are expected to be involved in molecular function, 16 in cellular components, and 38 in biological processes (Supplementary Figure 6A). Different KEGG systems were used for metabolic pathways prediction of gene products and compounds. Further, orthologous groups of proteins were predicted by protein database COG, which predicted eight functional genes (Supplementary Figure 6B).

Annotations were carried out by The NR (Non-Redundant), a protein database that gave us complete species information; the bacteriophage genome resembled about 14 different bacterial species (Supplementary Figure 6C). Then, TCBD functional classification showed that it has two proteins, one is holin (MSK_000044), and the other one is putative transport protein (MSK_000046) (Supplementary Figure 6D). Another classification showed it has one channel pores, and the other is an incomplete transport system (MSK_000044, MSK_000046) (Supplementary Figure 6E). The enzyme search through The CAZy database showed that there was only one major Glycoside Hydrolases (GH) enzyme member in the sequence (MSK_000045), which functioned as a Lysozyme RrrD as shown in Supplementary Figure 6F. Lysozyme is the hydrolyzing enzyme that causes the lysis of the 1–4 beta-linkages between N-acetylmuramic acid and N-acetyl-D-glucosamine residues in a peptidoglycan and between N-acetyl-D-glucosamine residues in chitodextrins. Particularly this enzyme helps the phage to release its progeny from the host cell by cytolysis. This enzyme can now also be used as an antimicrobial and bacteriolytic for therapeutic purposes because of its hydrolyzing ability against pathogenic bacteria (Srividhya and Krishnaswamy, 2007).

Proteins generally contain functional regions called domains, so predicting protein domains for analyzing protein function was done through Pfam (Supplementary Table 1A). Moreover, Pfam clan; Pfam families emerge from a single evolutionary origin (Supplementary Table 1B). After that high level of annotation, results like function, domain structure, post-translational modification, and variations are achieved through the swiss-pro database, which is mentioned in Supplementary Table 2.

The secreted protein synthesized in the cell and secreted out of the cell through the cell membrane under the guidance of a signal peptide composed of acid. Whereas the secreted protein N-terminus is composed of 15–30 amino groups. Six transmembrane helicase encoding regions predicted by TMHMM in some genes (MSK_000011, MSK_000018, MSK_000044, MSK_000046, MSK_000050, MSK_000067), while only two secretory signal peptide encoding genes were determined by SignalP in genome (MSK_000011, MSK_000046). The result obtained is also shown in the table (Supplementary Figure 7A).

Among the 73 predicted genes, no single gene was identified in the type N secretion system, which helps find a pathological response (Supplementary Figure 7B). EffectiveT3 software (Version 1.0.1) prediction shows that there only 4 true T3S effector proteins (MSK_000026, MSK_000028, MSK_000031, and MSK_000052) and 69 are false T3SS effector proteins (Supplementary Figure 7C). During the genome, annotation results showed no secondary metabolites, and pathogen-host interaction was founded.

Then, features analysis showed that there are 73 open reading frames in assembled MSK phage genome. Among these 73, 49 were called hypothetical proteins, as no homologous with proved function exists in the genome database. Twenty-four showed a close resemblance with identified functional proteins, divided into five putative functional protein modules; Morphogenesis proteins, metabolic enzymes, lysis cassette, additional phage feature proteins, and hypothetical proteins.

Genome analysis revealed that there were at least 16 ORFs involved in the morphogenesis of bacteriophage MSK. These proteins include major capsid protein, portal protein, lipoprotein, tail fiber, tail assembly and tail tap measure proteins, tRNA, and DNA packaging proteins (terminase large and small subunit); among these proteins, tail fiber protein and lipoprotein gave a close homology with Rtp bacteriophage. In addition, we identified seven ORFs that were involved in the metabolism, including primase and helicase, recombinase, DNA adenine methyltransferase, polynucleotide kinase/phosphatase; these enzymes aid in nucleotide metabolism. Due to the presence of the primase and helicase, the phage is independent of a host and uses its machinery for replication. In addition, phage MSK recombinase (MSK_000027) is considered to play a role in genome repair, replication, and recombination.

**FIGURE 6:**
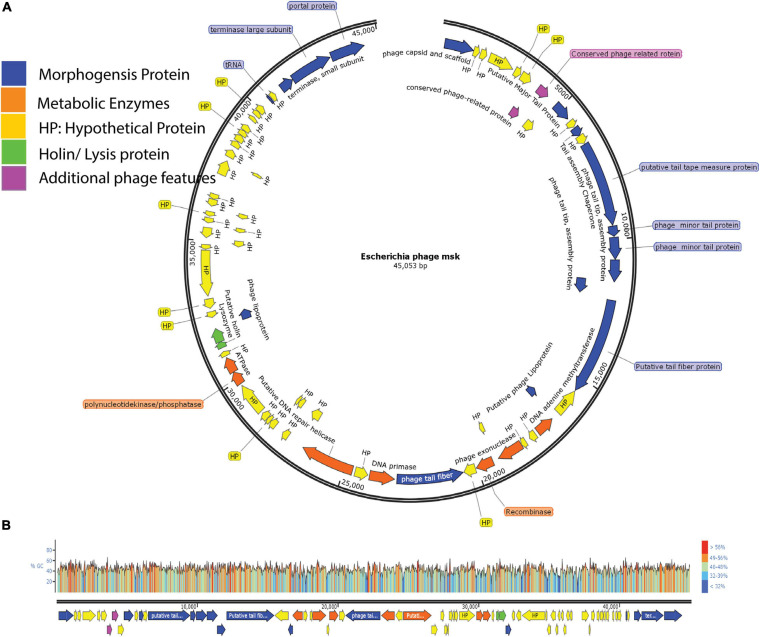
Genome Map of *E. coli* phage MSK and GC content percentage lies in whole-genome. **(A)** The genome map of phage displays putative ORFs divided into five different modules with unique colors. Arrow direction of each ORF indicating the transcription direction, **(B)** G + C content of the phage genome lies between 40.8%, and colors in the GC content shows the percentage ratio.

We also identified two ORFs encoding proteins associated with lysis cassette (holin and lysozyme), responsible for the host cell lysis. Among these, protein holin helped to form a pore and provide a channel for lysozyme to access and degrade the bacterial cell wall (peptidoglycan layer). Besides, two additional phage proteins helped the phage in morphogenesis or metabolism. Finally, these four feature modules and 24 hypothetical proteins are explained in the feature table (Supplementary Table 3). ORF with the locus tag MSK_000019 encodes the most significant protein (putative tail fiber proteins) with a size of ∼ 277 kDa. ORF with the locus tag MSK_000036 encodes the smallest protein (hypothetical protein) with a size of ∼10.13 kDa. The orientation of the genome shows that most of the genes (55) are on the plus strand, while 18 are on the reverse strand. The linear genome map of all hypothetical and functional proteins groups and their GC content percentage is presented in Figures 6A,B.

### Analysis of Virulence and Antibiotic Resistance Genes

Another essential factor to be measured in bacteriophages is the virulence and the presence of antimicrobial-resistant genes. Nowadays, antimicrobial resistance (AMR) is becoming a global threat to public health and food security as it might cause about 10,000,000 deaths per year by 2050 (O’Neill, 2014). Besides, the world is looking for new antimicrobial referents that can substitute the old ones. To overcome this problem, bacteriophages are an auspicious choice against these superbugs (MDR and XDR). But, unfortunately, phages also act as a vehicle for resistance and virulence gene transmission, which further cause the spread of antibiotic resistance (Gómez-Gómez et al., 2019). Previously, in 2012, a phage with a Shiga toxin (*stx*) was confirmed to regulate the antibiotic resistance gene (Granobles Velandia et al., 2012).

Presently, the lifestyle of bacteria predicted that Phage MSK is a lytic phage and kills its host within 2 h at MOI = 10, with no lysogenic genes (Supplementary File 3). Temperate bacteriophages can share their genome with the host, which might cause the spread of antibiotic resistance and virulence gene transfer in phage therapy. Furthermore, bioinformatics analysis was done to find the antibiotic resistance gene, but there was no significant hit based on AMR (anti-microbial resistance) gene identified in the complete genome. Similarly, for virulence genes, the analysis VirulenceFinder 2.0 predicted that the MSK genome did not contain any virulence genes. Therefore, we assumed that MSK could be further used for phage therapy against drug resistance coliphages through these findings.

### MSK Phage Protein Profile Prediction

**FIGURE 7:**
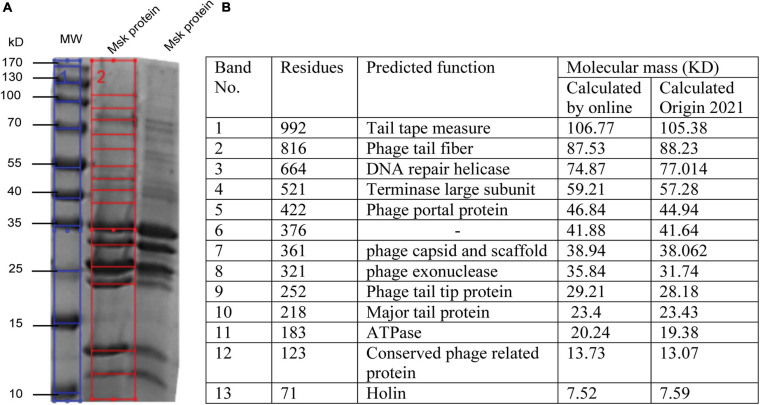
SDS PAGE and *In silico* analysis of structural protein of phage MSK. **(A)** Coomassie blue staining of a 12% SDS–polyacrylamide gel showing structural proteins from phage MSK. (1) Blue line: The sizes (kDa) of different proteins from the broad-range weight marker (MW) are indicated on the left side (2) Red line on the right indicates the bands identified from the gel by Gel molecular weight analyzer tool [Origin(Pro), 2021] and verified by online molecular weight analyzer. **(B)** Identification of MSK various proteins and their predicted function from corresponding bands shown in the table.

Further, the structure profile of bacteriophages is determined by SDS-PAGE, enabling us to determine the individual protein, molecular weight, and size. These analyses can be used for the morphotyping and differentiation of distinct proteins even within the same species (Urban-Chmiel et al., 2018). ORF prediction revealed that fewer genes are encoding structural proteins. The structural protein of phage MSK was characterized by SDS-PAGE coupled with a gel molecular weight analyzer (Origin(Pro), 2021). Purified phage particle was heated and run on 2-D SDS-PAGE, and protein bands were analyzed using coomassie staining. Further, the protein SDS_PAGE picture was brought to the Gel molecular weight analyzer, which calculates thirteen bands using regression value (Figure 7B). That leads to the identification of morphogenesis, holin, metabolic enzymes, and additional phage feature protein (Figure 7A).

Molecular weight, calculated by Origin(Pro), 2021, was compared with a phage MSK coding region with the same molecular mass (kDa) calculated online predicted different function (Figure 7B). The molecular weight of 13 phage MSK structural proteins was depicted on SDS-PAGE ranging from 7 to 106 kDa representing MSK structural proteins. The most predominant protein was band #10, considering the morphology of this phage, MSK_000010, which is the Major tail protein. Other proteins having the locus tags MSK_000017, MSK_000025, MSK_000042, and MSK_000044 were also detected with high coverage, but the number of detected peptides was less in comparison with MSK_000010. Overall, the molecular masses estimated by SDS-PAGE for most proteins were in agreement with the calculated masses from the gene sequence (Figures 7A,B).

Phage MSK structural profile possesses several features. The structural protein MSK_000014 is (tail tape measure protein) the most significant gene having 992 amino acid residues while MSK_000019 tail fiber protein is the subordinate structural gene having 816 a.a (amino acid), a characteristic shared by many tail fiber phages; these genes are located upstream of minor tail protein, tail assembly protein. Both of these proteins show amino acid sequence similarity to several tail phages. Thirteen MSK structural proteins were identified by SDS-PAGE analysis, including 12 major structural proteins. Among these proteins, terminase large subunit and others which were similar were involved in the pac-DNA packaging strategy encoded in the genomic region of MSK. This region is homologous to the terminase of the other *E. coli* phages suggesting a horizontal gene transfer between the phages and host and contributes to genetic diversity and evolution. Therefore, there is an immense desire to determine the sequences of phages because the phage genomics provides the phage and bacterial evolution tracing.

### DNA Packaging Strategy and Phylogenetic Analysis

DNA packaging strategy is another most essential component of phage structural analysis. The terminase small and large protein units play a role in the initiation, termination, and translocation of the phage DNA into proheads (Fujisawa and Morita, 1997). In this regard, MSK bacteriophage has two genes (MSK_00071 and MSK_00072) encoding terminase small and terminase large subunits; usually, both of these genes are adjacent like in Rtp and T1 (Roberts et al., 2004). The role of the small terminase is DNA-binding activity, and the large terminase provides ATP binding, prohead binding, and DNA cleavage activities (Wietzorrek et al., 2006). The phageTerm analysis showed that genome packaging of the *E. coli* phage MSK took place through headful packaging using pac-site on concatemeric precursor which lies in type unknown class; the exact mechanism was already identified in phage Rtp and T1 and phiC19 (Ramsey and Ritchie, 1983; Wietzorrek et al., 2006; Amarillas et al., 2016). From previous results, we can predict headful packaging in MSK bacteriophage occurring in the forwarded direction. The phageTerm method showed that it starts from position 36,862 in the genome, having a *p*-value (1.62e^–09^), and Peaks localized 20 bases around the maximum. Li’s method showed unique termini on the forwarding strand and no obvious termini on a reverse strand. The Phage genome does not have any termini and is either circular or completely permuted and terminally redundant, as shown (Supplementary File 2; Garneau et al., 2017).

In the same manner, NCBI blast results suggested that both large and small terminase have very close homology to Rtp and DTL bacteriophages which were previously isolated from industrial *E. coli* through a fermentation process in Germany and the United States. Further, finding the pac-site in the genome, MSK was compared with closely homolog bacteriophage Rtp. Wietzorrek et al. (2006) previously predicted five repeats of the ATATA sequence in T1 phage and closely related phage. However, genome analysis of phage MSK showed that these motifs were also not present in the genome, similar to Rtp.

**FIGURE 8:**
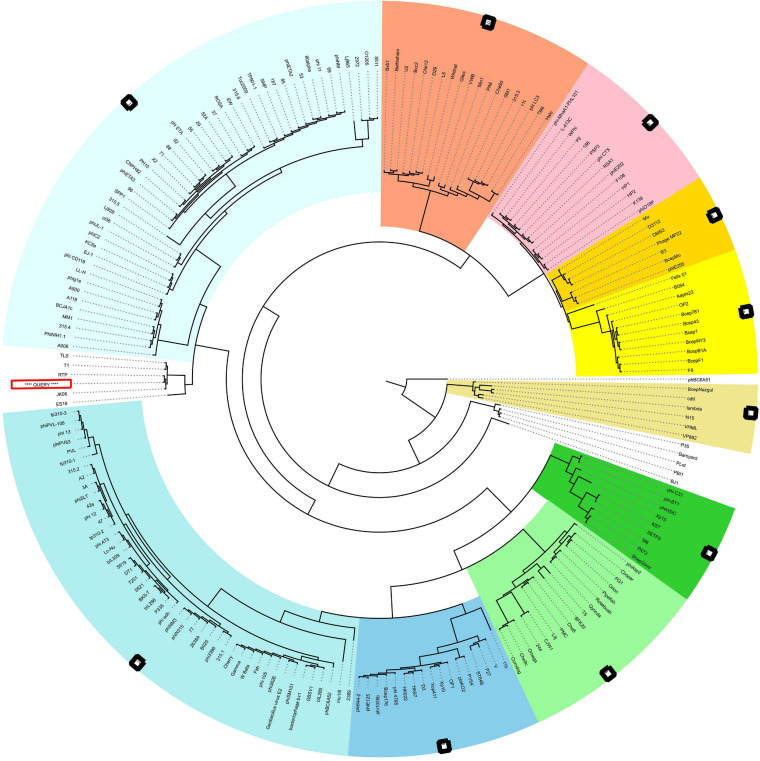
VIRFAM Tree analysis of MSK bacteriophage. VIRFAM generated clustering of MSK with phages sharing the most identical neck-head-tail modules. Different type 1 phage clusters are highlighted with different background colors. Phages of type 1 with no cluster assigned are highlighted with white background. MSK phage is indicated by the red box (Query sequence).

The neck, head, and tail structure genome organization of phage MSK phage belong to *Siphoviridae* of type 1 (no cluster assigned) similar to Rtp. Interestingly, the TEM micrograph performed on the sample obtained by iodixanol gradient showed that structures compatible with Rtp phage head, tail, and tail fiber were witnessed (Figure 2H). We also observed that genome coverage of the sequencing was evenly distributed over the entire genome and share 47 ORF with Rtp bacteriophage. Along with Rtp, phage MSK closely resembles no other cluster assigned phages (T1, TLS, JK06, and ES18) (Figure 8). Phage MSK structural genes like (head, neck, and tail) have similar sequence and function with known Rtp phage and classify the phage with respect to other phages in Aclame (a classification of Mobile genetic Elements). These findings also support the phage tail module (terminase large protein) having close homology with Rtp bacteriophage and T1 bacteriophage, which has predicted the headful DNA packaging mechanism.

The Viral Proteomic tree analysis revealed that phage MSK protein sequences are almost similar to the bacteriophage RTP. ViPTree, dendrogram reveals global genomic similarity relationships between 579 bacteriophage sequences of different families. These relationships predict RTP bacteriophage and MSK falls in *Siphoviridae*. But according to the Rtp lineage information provided in the genome database (GenBank) the phage belongs to *Drexlerviridae* family. These findings concluded that phage MSK belongs to the *Drexlerviridae* and subfamily *Braunvirinae* (Supplementary Figure 8).

### Comparative Genome Analysis of MSK Phage With Closely Related Phages

MSK comparative analysis was done with four *E. coli* phages [phage vB_EcoS-2862V (MK907276.1), *Escherichia* phage vB_Ecos_CEB_EC3a (NC_047812.1), *Escherichia* phage DTL (NC_047893.1) and, *Rtp* bacteriophage (AM156909.1)], by calculating the average nucleotide identity (ANI). ANI is widely used to compare the prokaryotic genome using USEARCH instead of BLAST. These four bacteriophages were isolated from different samples and shared ANI of 87.18, 85.32, 83.97, and 84.79 %, respectively. BLASTn of the phage MSK with Rtp showed that 89.60% of the MSK genome was homologous to the Rtp genome, covering almost entirely the functionally annotated region of the phage Rtp genome, the closest homology of the MSK genes are found in the Rtp phage as shown in the table (Supplementary Table 3).

**FIGURE 9:**
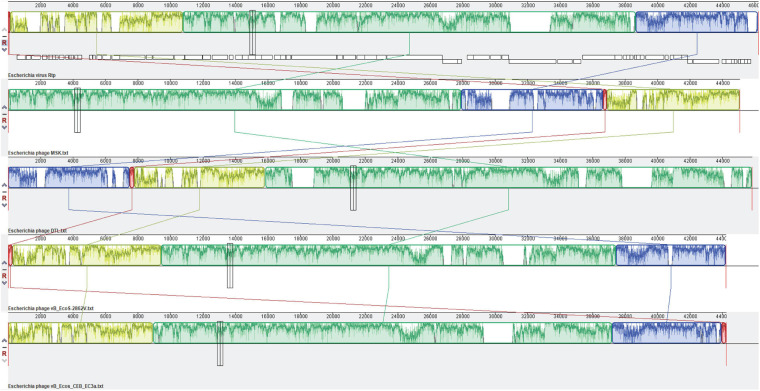
Comparative genomic analysis of *Escherichia coli* phages of the *Drexlerviridae* family. The genome map of phage MSK displays putative ORFs shown with different colors and arrows indicating the transcription direction. The colored bars indicate homologous DNA regions between phages (Rtp, phage MSK, DTL, and B_EcoS-2862V). The connection lines indicate homologous regions between the genomes of four *Escherichia* coliphages. Rtp bacteriophage genome was downloaded from the NCBI genome database and used as a reference sequence.

The genome comparison with Rtp bacteriophage showed that it has 26 genes similar to MSK phage. Comparing the morphological features of the bacteriophage *Rtp* has a close resemblance to a rosette-like tail, and its tail fiber protein (MSK_000019) gave 95.92% homology with the MSK phage. The custom database was used for subsequent comparative analysis. First, a multiple sequence alignment was performed; a comparison of the relatedness of the different nucleotides sequences was generated utilizing Mauve (Darling Lab). The genomic comparison of the phage MSK with *Escherichia* phage vB_EcoS-2862V, *Escherichia* phage vB_Ecos_CEB_EC3a, *Escherichia* phage DTL and, Rtp bacteriophages are shown (Figure 9). These results suggested that these phages have extensive synteny between genome structure with percentage identity 91.18, 88.44, 85.37, and 89.48% among these bacteriophage genomes. But it also shows some mosaicism in the genome, including ORF which encodes tRNA. The tRNA-Arg encoding gene was not present in bacteriophage *Rtp* and DTL but present in the *Escherichia* phage vB_EcoS-2862, *Escherichia* phage vB_Ecos_CEB_EC3a. These findings concluded that phage MSK belongs to the *Drexlerviridae* family and subfamily *Braunavirinae* and virus belong to *Rtp* viruses.

### Antimicrobial Activity Against Multidrug-Resistant Coliphages

The prevalence of antibiotic resistance (AMP, TCN, and Colistin) and extended-spectrum beta-lactamase (ESBL) production has considerably escalated in all terrestrial regions. Infections due to these c bacteria are associated with high mortality across human beings. Phage MSK can kill pathogenic bacteria. That offers a novel approach with an unprecedented and orthogonal mechanism of action for the treatment of diseases.

A panel of five *E. coli* phages obtained from Zhejiang university hospital and labs mentioned in the table were used to test the infectivity of the newly discovered MSK phage using a spot test. Overall, MSK bacteriophage demonstrated a consistent narrow range; infecting 100% of clinical multidrug-resistant strains of *E. coli* were tested. But, this novel phage was unable to infect the *Salmonella* and *Pseudomonas* species. These findings concluded that this phage could only be used against drug resistance *E. coli* bacteria. All strains were susceptible to MSK phage gave plaques clear to evident plaques formation on LB agar plate (Table 1).

**TABLE 1:** Antimicrobial activity against multidrug resistant bacterial strains.

Name of bacterial strains	Source of strains	Activity/spot assay
*E. coli* (AMP)	Zhejiang University First Affiliated Hospital, Hangzhou	+
*E. coli* (ESBL)	Zhejiang University First Affiliated Hospital, Hangzhou	+++
*E. coli* (TCN)	Zhejiang University School of Medicine (C602)	+++
*E. coli* MG1655 (Colistin Resistant, NMCR-1	Department of Pathogen Biology and Microbiology	+++
*E. coli* MG1655 (Colistin Resistant, MCR-4	Department of Pathogen Biology and Microbiology Zhejiang University School of Medicine	+++
*Salmonella anatum* (Epsilon 15 host)	Department of Biochemistry and Genetic, Zhejiang University School of Medicine	−
*Pseudomonas syringae* (Phi-6 host)	Department of Biochemistry and Genetic, Zhejiang University School of Medicine	−

*Escherichia coli, *E. coli*; AMP, Ampicillin; ESBL, Extended Spectrum Beta-Lactamase; TCN, Tetracycline; NMCR-1, Non-mobile Colistin Resistance; MCR-4, mobile colistin resistance; +, Clear plaque; +++, very clear plaque; −, No plaque formation.*

## Discussion

Bacteriophages are the most abundant biological entities present in all environments (natural and artificial) with their bacterial communities. These phages might have positive or negative ecological impact by eradicating recalcitrant bacteria from the natural environment (Meaden and Koskella, 2013; Batinovic et al., 2019). Several bacteriophages have been isolated previously from the natural environment (human) (Pacífico et al., 2019) and artificial environment (industrial units) (Wietzorrek et al., 2006; Halter and Zahn, 2018). This study characterizes the coliphage phage MSK, which explains the phages from the naturally occurring environment. Phage MSK can infect *E. coli* C, Previously temperate phage P2 and lytic phage X 174 have been isolated from the common host, despite no conserved proteins. Phage morphology and genome type were identified among these *E. coli* phage’s genomes (Wiman et al., 1970; Barreiro and Haggård-Ljungquist, 1992; Zheng et al., 2009). Bacteriophage MSK shares identity features with the coliphages community, including morphological resemblance and genome size. These phages consist of a dsDNA genome with a size range of 45–50 kb bp and GC content of about 45–50% (Roberts et al., 2004; Pacífico et al., 2019). MSK bacteriophage showed 91% nucleotide sequence identity to 35 Rtp like Escherichia coliphages isolated from the clinical samples (Pacífico et al., 2019). Rtp phenotypes bacteriophages isolated from industrial and other animal sources also showed 85 to 90% nucleotide identity features (Wietzorrek et al., 2006; Ferreira et al., 2020). NCBI blast result showed that Escherichia phage vB_EcoS-2862II (MK907273.1) has the highest percentage identity (91.18%), Rtp bacteriophage (89.48%; AM156909.1), and DLT (85.37%; NC_047893.1).

Inevitably, there are many studies showing phages are present where bacteria reside. But, now, this hypothesis may not work because phages are isolated where bacterial abundance did not exist. Previous studies showed that these Rtp like phages could be isolated from the body fluid, as the human body is also not sterile (Pacífico et al., 2019) and many industrial units (Wietzorrek et al., 2006; Halter and Zahn, 2018) that can cause many problems in terms of quality, quantity, and an experimental result which further causes finical issues (Halter and Zahn, 2018). We noticed several others phage prevalence in the lab environment, including coliphages and *Pseudomonas* phage. Our lab isolated three morphological different pseudomonas phages (Data not shown here). We assume that some bacteriophages present in the lab environment, which might need to increase the host range, can further help to increase the number of phage identification.

The lifestyle of the phage MSK, like other T1 phages, demonstrates that it is a lytic phage with the ability to reduce the growth of *E. coli* C during 4 h of infection (Figure 3). Consequently, the genome of phage MSK has a holin gene (MSK_000044) right upstream of the lysozyme gene (MSK_000045). This constitutes its lytic cassette, indicating that the lytic activity of the lysozyme is holin-dependent and produces larger plaques and larger burst size (Abedon and Culler, 2007). In addition, our isolated phage MSK showed a latent period and burst time of 25 and 45 min, respectively. In comparison, *Drexlerviridae* family phage phiC119 showed a latent period of 20 min and burst size (210) 58 min (Amarillas et al., 2016). In contrast, T1 bacteriophage exhibit a latent period of 13 min and produces 100 virions per infected phage (Borchert and Drexler, 1980). Further, several factors like mutation, host cell size, host physiology, and culture condition might reflect the short burst size and the latent period during the propagation in the host (Sharaf et al., 2018). These results suggest that the latent period of the phage MSK is almost closed to other *Drexlerviridae* family bacteriophages, and this could be a potential candidate for phage therapy.

Physiochemical characteristics like temperature, pH, and UV might affect the activity and stability of phages (Jończyk-Matysiak et al., 2019). In this regard, bacteriophage MSK demonstrated stability to high temperature (100°C), pH, and UV exposure and did not adversely affect phage survival; yet, stability was not observed at 120°C. Maximum stability of phage MSK was observed at 100°C with a decrease in plaque size (Supplementary Figure 2B). Many studies reported that bacteriophages are commonly stable at the temperature range (60–70°C), and a temperature rise may decrease the number of plaque-forming units (Ramírez et al., 2018; Alvi et al., 2020). Similarly, phages are sensitive to acidic media; our results showed that phages are resistant over a wide range of pH. In general, the natures of phages make them sensitive to UV-irradiation by reducing their concentration or inactivating phage infectivity (Clark et al., 2012).

Interestingly, phage MSK infectivity was not decreased at various temperatures and UV exposure, as discussed earlier. On the contrary, previous studies reported where UV-light is used to cause mutation in bacteriophages to increase or decrease the host range (Kadavy et al., 2000). Conclusively, phage MSK showed stability against UV and heat treatment and did not show any alterations in plaque forming unit (*PFU*).

The genome of the phage MSK comprised 45,053 base pairs with a GC content of 44.8%. This GC content value was lower than its host which is consistent with previously reported phage phiC119 (Amarillas et al., 2016). Usually, lytic phages have lower GC content than their host, in contrast to temperate phages having an equal percentage to GC content (Rocha and Danchin, 2002; Amarillas et al., 2016). Furthermore, genome size determined 73 ORFs in phage MSK which are almost familiar with *Rtpvirus* (Rtp, DTL). The size of bacteriophage MSK is compared to those of the *Rtpvirus* genus bacteriophages Escherichia phage vB_EcoS-2862V (44,219 bp) and Escherichia phage vB_EcoS-2004IV (44,219 bp) (Pacífico et al., 2019). The phage MSK genome also shares a characteristic of overlapping ORFs with other phages, which enable the phage to minimize the genome size and play a role in regulating gene expression (Johnson and Chisholm, 2004; Pavesi, 2006).

Bioinformatics analysis of the bacteriophage MSK genome revealed that the genome is organized into five different functional components, showing the genome is arranged in a compact manner (Figure 2). Like other tailed bacteriophages, these modules performed different biological functions in phage morphology, metabolism, lysis, DNA replication, and repair (Amarillas et al., 2016). Thus, the phage MSK has a mosaic genome structure and morphology similar to related phages, indicating the extensive genetic exchange and horizontal gene transfer within the phage community (Rohwer and Edwards, 2002). Furthermore, the phage genome also has the one tRNA-arg (78 bp), which also supports the hypothesis that bacteriophages with tRNA genes have larger burst size as a result of their virulence and propagation in their host increases (Delesalle et al., 2016; Sharaf et al., 2018).

MSK bacteriophage genomic region MSK_000019 encoding the module of the distal part of its tail that is over 90.99% identical to the gene segment of several Escherichia coliphages. This open reading frame is 90% homology with Rtp 43 protein (CAJ42247.1) involved in the host cell recognition (Wietzorrek et al., 2006). The metabolic module of the phage MSK consists of DNA primase and helicase, suggesting that phage replication is independent of the host machinery (Figure 2). Moreover, lipoproteins having N-terminal are also valuable contributors in sorting signals and playing roles in preventing superinfection by inactivation of receptors (Tokuda and Matsuyama, 2004). Interestingly, we also predicted a lipoprotein in the phage MSK genome which showed homology and had a conserved domain with Rtp 45 and T5 lipoproteins (Braun et al., 1994; Tokuda and Matsuyama, 2004; Wietzorrek et al., 2006).

Virulent and temperate bacteriophages have contained different recombinase families. There is six families of recombinases have been identified so far. Virulent and temperate bacteriophages contain two and four families of recombinases, respectively (Lopes et al., 2010). Virulent MSK phage genome might encode Gp2.5 recombinase (Erf), because similar recombinase has been identified in Rtp, T1, and T7 bacteriophage (Wietzorrek et al., 2006). MSK recombination gene (MSK_000027) is located (19403–20053) on the top strand, upstream of the phage exonuclease gene (MSK_000025). NCBI BLASTn analysis showed that MSK phage recombinase shares 97.69% homology with Rtp bacteriophage (Rtp49). These findings assumed that recombinase (MSK_000027) might play an essential role in replication, genome repair, and recombination facilitated by single-stranded DNA binding protein (Ssb) as experimentally verified in T7 bacteriophages (Kong and Richardson, 1998).

DNA packaging and its mechanism are also of prime importance in the lifestyle of a phage. In this regard, it is observed that phage MSK has a headful packaging strategy (pac-mechanism) of the type unknown already described for related bacteriophages, such as Rtp, phiC119 (Ramsey and Ritchie, 1983; Wietzorrek et al., 2006; Casjens and Gilcrease, 2009; Amarillas et al., 2016). Furthermore, a classification based on the structure modules (head, neck, and tail) was conducted, which indicates that the phage tail module (terminse large protein) was homolog with Rtp phage. These findings suggested that the phage MSK packages its DNA by headful packaging mechanism (pac-sites) similar to that of Rtp and T1, resulting in terminal redundancy and circular permutation. Furthermore, previous studies showed that phage phiC119 use the same packaging strategy as T1 based on homology (Ramsey and Ritchie, 1983; Wietzorrek et al., 2006).

Comparative analysis of the entire genome sequences of the *Escherichia* phages B_EcoS-2862V, B_Ecos_CEB_EC3a, RTP, DTL, and MSK was done to characterize the prophage. Among these phages, MSK bacteriophage revealed the highest correspondence (87.18% ANI) with Escherichia coli phage B_EcoS-2862V. As the Rtp bacteriophage is well studied in terms of the genome, phage MSK also showed higher diversity (84.79% ANI) in these phages. These identified sequences with the highest ANI values can be used in evolutionary and phylogenetic studies, particularly when the whole genome sequence is not available in the database. In addition to showing a robust evolutionary relationship, the whole-genome nucleotide MAUVE plot also showed a higher homology region among these phages of the same family. These local homology regions are supposed to be recombination sites and conserved regions among these lytic phages (Figure 9). Close comparative genome analysis of the phage suggested that it could be used in the antimicrobial resistance.

Like many other phages, MSK showed negligible intraspecies host range (*Salmonella, Pseudomonas*), while interspecies host range was 100%. This phage could infect the *E. coli* laboratory strains (DH5α and Rosetta and *E. coli* top 10). The presence of the specific host cell receptor provides the specificity in targeting the host, which can be further helpful for treating coliphage-related infections like ampicillin, tetracycline, and Colistin resistance (MCR-1 and MCR-2) *E. coli* bacteria (Table 1).

These observations highlight the importance of using phages in therapy. These findings ensure that the phage is strictly lytic and does not code any antibiotic-resistant and virulent genes. We know phages are a powerful tool for phage therapy, but if phages got resistant to bacteria, this might impede their potential application in phage therapy and biocontrol (Oechslin, 2018). Complete bioinformatics analysis revealed that bacteriophage MSK did not contain lysogeny proteins. Therefore, no undesirable antibiotic resistance genes and virulence genes were present but in the phage genome. These results suggested that phage MSK might be safe at the genome level and could be used in phage therapy.

In summary, genome analysis and electron microscopy revealed that phage MSK belongs to the *Derxivirdiae* family. Furthermore, phages exhibit a short latent period with a broad host range against coliphages, a narrow host range against other bacterial species, and stability at higher temperatures (100°C) and pH ranges. Genomic studies suggested that phage did not contain any antibiotic resistance and virulence genes. These results indicated that phage MSK might be suitable for phage therapy against antibiotic resistance of bacteria. In addition, phage MSK demonstrated strong lytic activity against Colistin resistance bacteria. However, further toxicity studies are required to ensure the safety of the phage. Therefore, our future research will be aimed at characterizing this phage for a better understanding of its potential as a biocontrol agent.

MSK bacteriophage belongs to the *Drexlerviridae* family of the *Caudovirales* order, *Rtpvirus* genus; it has a large icosahedral capsid and a long tail with four short linked rosette-like morphology of the tail tip. This phage has a morphotype similar to Rtp bacteriophage, which we assume may be responsible for host recognition.

## Data Availability Statement

The datasets presented in this study can be found in online repositories. The names of the repository/repositories and accession number(s) can be found in the article/Supplementary Material.

## Author Contributions

JZ acquired the funding and managed the project. JZ and MK conceived the study and designed the experiments. MK performed the experiments. SM, KL, and XG assisted with the experiments. MK analyzed the data, performed transmission electron microscopic analysis, prepared the initial draft, and wrote the manuscript. SM reviewed and edited the manuscript. All authors contributed to the article and approved the submitted version.

## Conflict of Interest

The authors declare that the research was conducted in the absence of any commercial or financial relationships that could be construed as a potential conflict of interest.

## Publisher’s Note

All claims expressed in this article are solely those of the authors and do not necessarily represent those of their affiliated organizations, or those of the publisher, the editors and the reviewers. Any product that may be evaluated in this article, or claim that may be made by its manufacturer, is not guaranteed or endorsed by the publisher.

## Footnotes

1
http://crispr.i2bc.paris-saclay.fr/Server/

2
http://www.genome.jp/kegg/

3
http://www.ncbi.nlm.nih.gov/COG/

4
http://pfam.xfam.org/

5
http://www.ebi.ac.uk/uniprot

6
http://www.cbs.dtu.dk/services/TMHMM/

7
http://www.cbs.dtu.dk/services/SignalP/

8
https://blast.ncbi.nlm.nih.gov/Blast.cgi?PAGE=Proteins

9
https://www.ebi.ac.uk/Tools/hmmer/

10
https://toolkit.tuebingen.mpg.de/tools/hhpred

11
http://www.snapgene.com/

12
http://arpcard.mcmaster.ca

13
https://cge.cbs.dtu.dk/services/VirulenceFinder/

14
https://galaxy.pasteur.fr/

15
https://www.genome.jp/viptree/
