# Supplementary material for: Virulent Drexlervirial Bacteriophage MSK, Morphological and Genome Resemblance With Rtp Bacteriophage Inhibits the Multidrug-Resistant Bacteria
Source: Front Microbiol. 2021 Aug 24;12:706700. doi: 10.3389/fmicb.2021.706700 (PMC8421802; doi:10.3389/fmicb.2021.706700)
Supplement: Supplementary file 1 [file Data_Sheet_1.pdf]

Supplementary Material

1 Supplementary Figures

1.1 Supplementary Figures 1

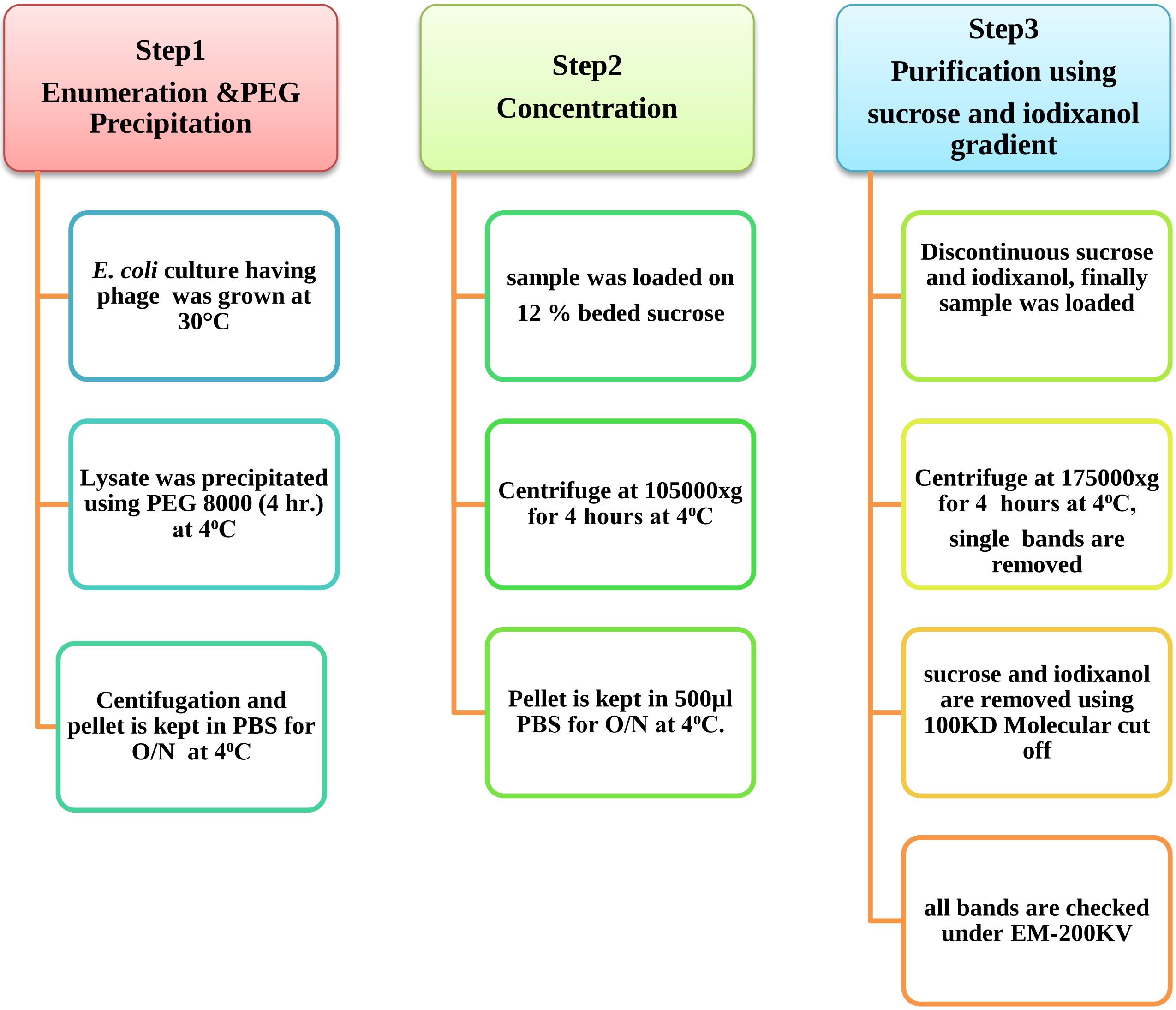

Supplementary Figure 1: Flow chart representation of phage MSK Enumeration and purification Protocol: These steps are following: step 1. Phage Enumeration and PEG precipitation, step 2. Concentration, step 3. Purification using sucrose and iodixanol gradient

1.2 Supplementary Figure 2

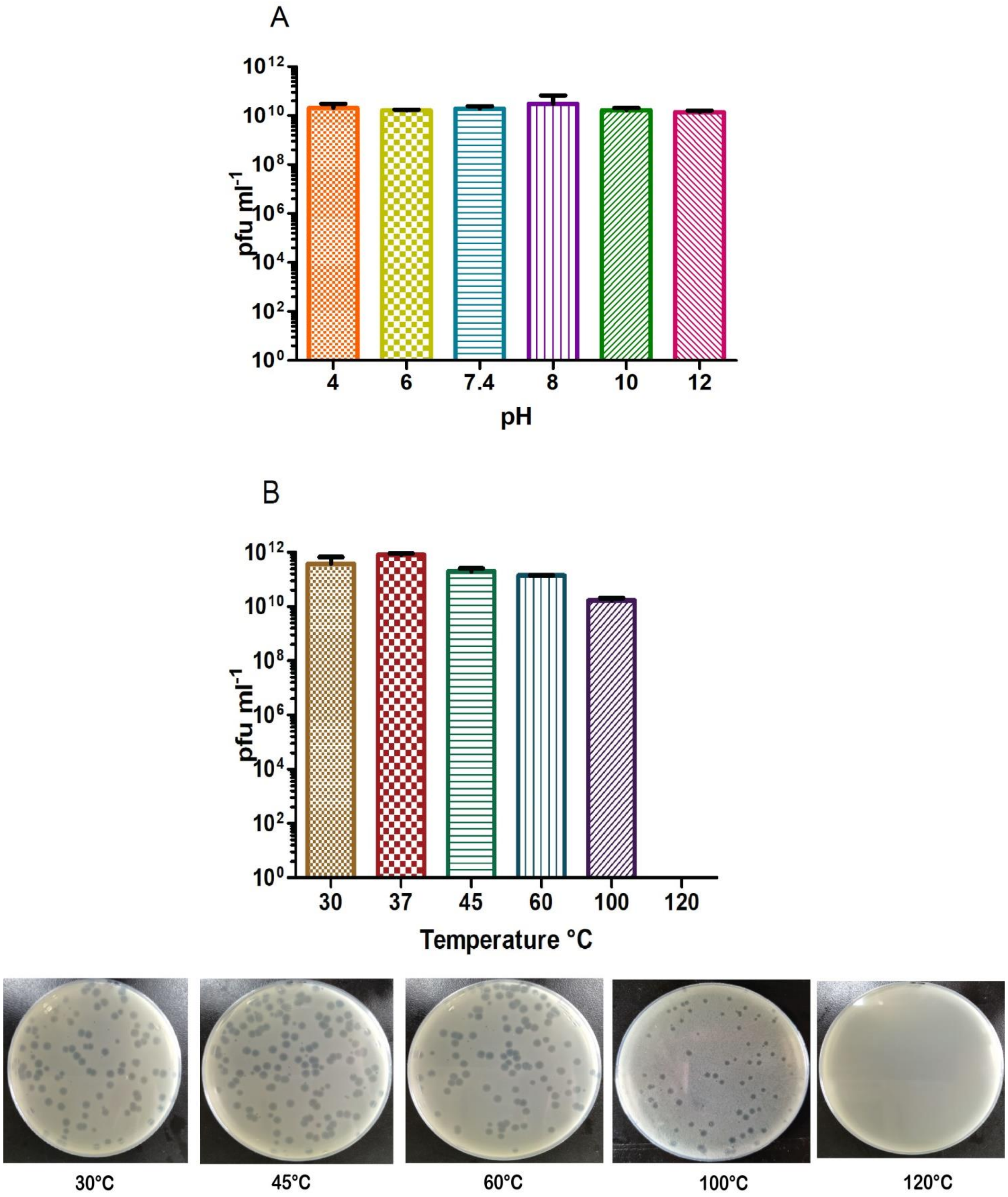

C

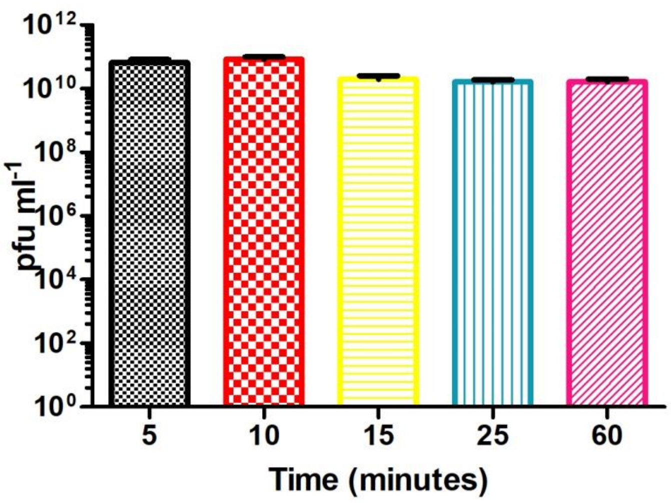

Supplementary Figure 2: MSK Phage stability under physiochemical conditions: (A) Effect of pH on the stability of MSK for 1 h at various pH at 37°C. (B) Effect of temperature on the stability of MSK after incubation for 1 hour at different temperature. (C) Effect of UV on the stability of MSK up to a period of 1 hour.

1.3 Supplementary Figures 3

A

Sample_name	1
Total Num(>500bp)	1
Total Length(bp)	45,053
N50 Length(bp)	45,053
N90 Length(bp)	45,053
Max Length(bp)	45,053
Min Length(bp)	45,053
Sequence GC%	44.19

C

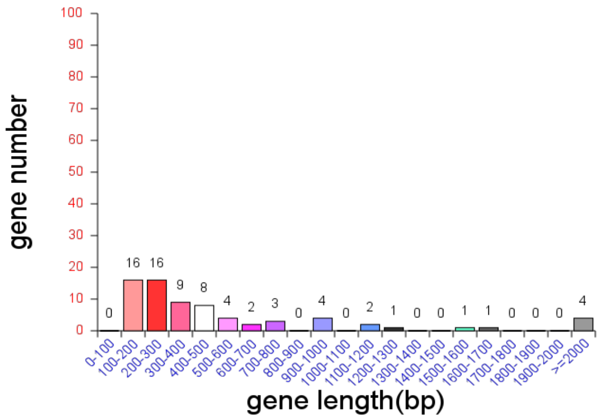

B

Genome Size:	45,053
Gene Number:	71
Gene Length:	40,053
GC Content:	44.8
% of Genome(Genes):	88.9
Gene Average Length:	564
Gene Internal Length:	5,000
Gene Internal GC Content:	39.39
% of Genome(internal):	11.1

Supplementary Figure 3: Scaffold sequence and coding gene estimation by different bioinformatics tools. (A) Scaffold sequence length estimated by SOAP denovo, SPAdes, ABySS, and gapclose. (B) Coding genes are estimated by GeneMarkS. (C) gene length distribution graph between gene number on Y-axis and gene length (bp) on X-axis.

1.4 Supplementary Figures 4

A

Type	Number(#)	Total Length(bp)	In Genome(%)	Average length(bp)
LTR	1	43	0.0954	43
DNA	1	61	0.1354	61
LINE	2	164	0.364	82
SINE	0	0	0	0
RC	0	0	0	0
Unknown	0	0	0	0
Total	4	268	0.5949	67

B

Type	Number(#)	Repeat Size(bp)	Total Length(bp)	In Genome(%)
TR	1	84~84	185	0.4106
Minisatellite DNA	0	0~0	0	0
Microsatellite DNA	0	0~0	0	0

Supplementary Figure 4: Repetitive sequence and tandem repeats sequence number estimation in the phage MSK genome. (A) Description of the table from left to right is the types of repetitive sequences (LTR: long terminal repeat sequence; DNA: DNA transposon; LINE: Long scattered repetitive sequence; SINE: Short scattered repetitive sequence; RC: rolling circle), number, total length (bp), % of the genome and the average length of repetitive sequence. (B) Description from left to right showing types of tandem repeats (TR: tandem repeat sequence; Minisatellite DNA; Microsatellite DNA), number, size, total length (bp) and % of the repeat sequence in the genome.

1.5 Supplementary Figures 5

A

Type	Number#	Avg_Len	Total_Len	% in Genome
tRNA	1	78	78	0.1731
5s	0	0	0	
16s	0	0	0	0
23s	0	0	0	

B

Prophage_ID	Locate	Start	End	Length	GC%
Prophage_1	Scaffold1	16781	38627	21847	42.4

Supplementary Figure 5: Non-coding RNA type and number estimation along with prophage sequence in the MSK genome. (A) Explanation from left to right are the type of ncRNA, the number of ncRNAs, the average length of ncRNA, and the total length of ncRNA. (B) From left to right are prophage ID, start and endpoint in the genome, the total length of prophage, and its GC content.

1.6 Supplementary Figures 6

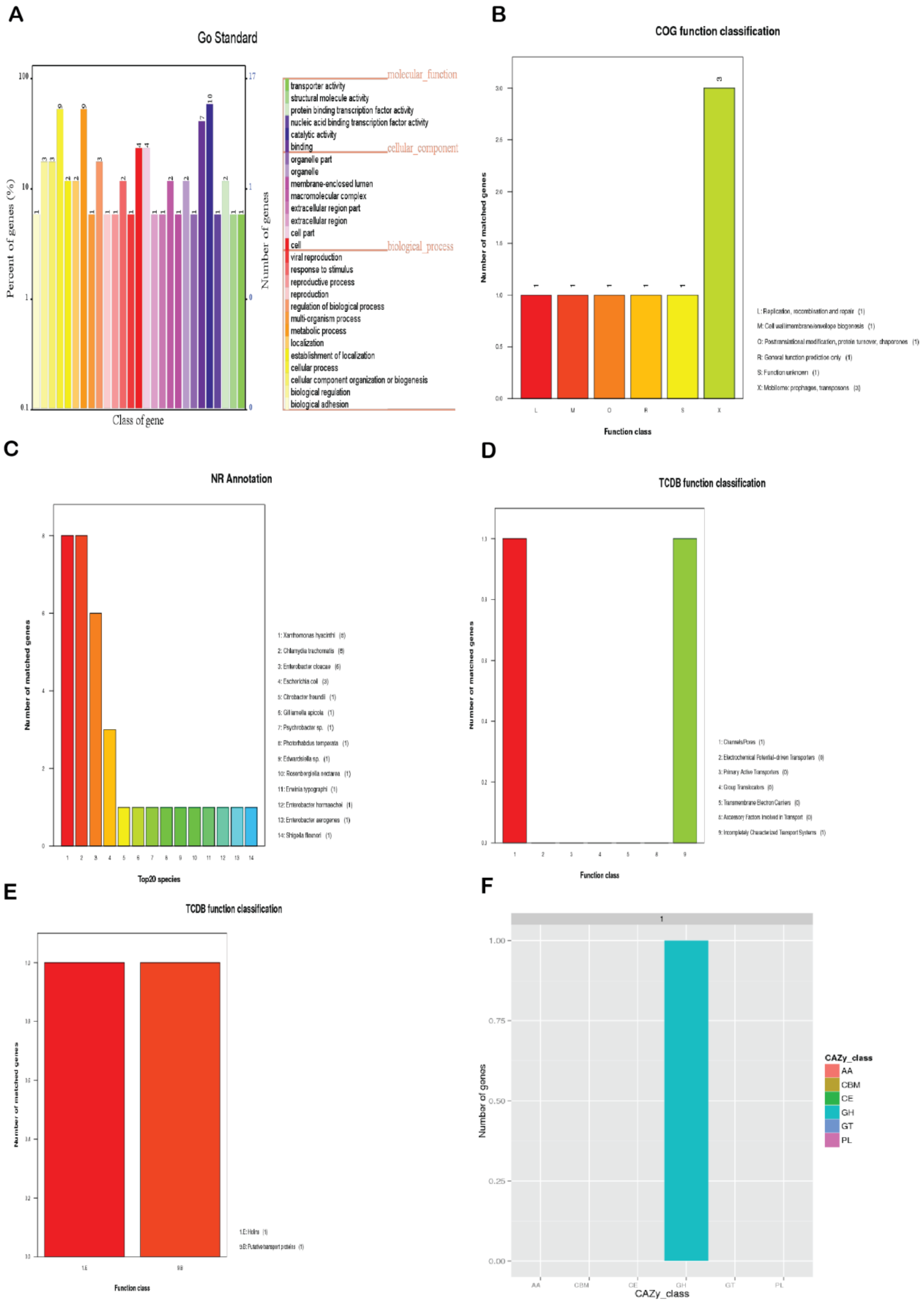
A
Go Standard
Percent of genes (%)
Number of genes
Class of gene
molecular_function
transporter activity
structural molecule activity
protein binding transcription factor activity
nucleic acid binding transcription factor activity
catalytic activity
binding
cellular_component
organelle part
organelle
membrane-enclosed lumen
macromolecular complex
extracellular region part
extracellular region
cell part
cell
biological_process
viral reproduction
response to stimulus
reproductive process
reproduction
regulation of biological process
multi-organism process
metabolic process
localization
establishment of localization
cellular process
cellular component organization or biogenesis
biological regulation
biological adhesion
B
COG function classification
Number of matched genes
Function class
L: Replication, recombination and repair (1)
M: Cell wall/membrane/envelope biogenesis (1)
O: Posttranslational modification, protein turnover, chaperones (1)
R: General function prediction only (1)
S: Function unknown (1)
X: Mobilome: prophages, transposons (3)
C
NR Annotation
Number of matched genes
Top20 species
1: Xanthomonas hyacinthi (8)
2: Chlamydia trachomatis (8)
3: Enterobacter cloacae (6)
4: Escherichia coli (3)
5: Citrobacter freundii (1)
6: Gilliamella apicola (1)
7: Psychrobacter sp. (1)
8: Photorhabdus temperata (1)
9: Edwardsiella sp. (1)
10: Rosenbergiella nectarea (1)
11: Erwinia typographi (1)
12: Enterobacter hormaechei (1)
13: Enterobacter aerogenes (1)
14: Shigella flexneri (1)
D
TCDB function classification
Number of matched genes
Function class
1: Channels/Pores (1)
2: Electrochemical Potential-driven Transporters (0)
3: Primary Active Transporters (0)
4: Group Translocators (0)
5: Transmembrane Electron Carriers (0)
8: Accessory Factors Involved in Transport (0)
9: Incompletely Characterized Transport Systems (1)
E
TCDB function classification
Number of matched genes
Function class
1.E: Holins (1)
9.B: Putative transport proteins (1)
F
Number of genes
CAZy_class
AA
CBM
CE
GH
GT
PL

Supplementary Figure 6: Statistics of gene functional annotation analysis of phage MSK using bioinformatics tools. (A) Abscissa represents gene function annotation GO function classification, the ordinate on the right represent the number of genes, and the ordinate on the left represents the percentage of coding genes. (B) The Abscissa indicate the type of COG function, and the ordinate indicate the number of genes in the annotation. (C) The abscissa indicates the species ID, and the ordinate indicates the number of genes in the annotation using NR database. (D, E) The abscissa indicates the first-level TCDB function classification, and the ordinate indicates the number of genes in the annotation. (F) The upper part is the sample ID, the abscissa is the classification type of the CAZy database, and the ordinate is the number of genes in the annotation.

1.7 Supplementary Figures 7

A

Sample ID	SignalP Protein(#)	TMHMM Protein(#)	Secreted Protein(#)
1	2	6	0

B

Sample ID	Total_Gene_Num	T1SS	T2SS	T3SS	T4SS	T5SS	T6SS	T7SS
1	71	0	0	0	0	0	0	0

C

Sample ID	Total Protein(#)	T3S effective true(#)	T3S effective false(#)
1	71	4	67

Supplementary Figure 7: Secreted protein and Secretion system identification in phage MSK ORFs. (A) The result obtained after running SignalP, TMHMM tools, from left to right, are the sample ID, number of protein with a signal peptide, number of protein with transmembrane and secreted proteins. (B) Type N secretion system was predicted by EffectiveT3 which shows not a single TNSS protein was recognized. From left to right be the sample IDs, the number of all coding genes and it is predicted to be the number of effector proteins of type I to type VII secretion system. (C) From left to right are the sample ID, the number of all coding genes, predicted as the number of T3SS effector proteins and predicted as non-T3SS effector proteins

1.8 Supplementary Figures 8

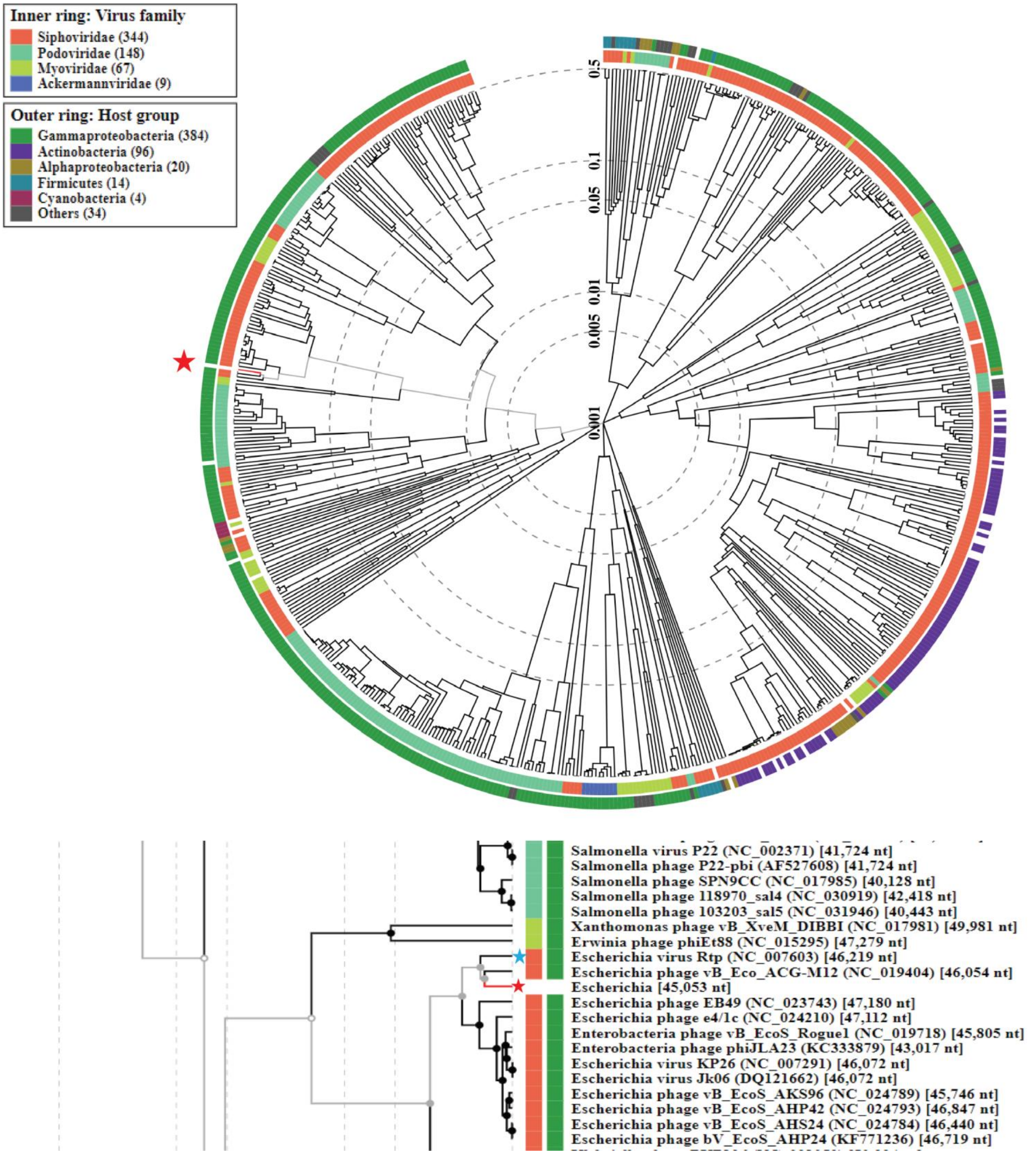

Supplementary Figure 8: Phage MSK phylogenetic tree constructed by ViPTree. ViPTree generated proteomics tree of phage MSK and 579 prokaryotic dsDNA viruses represented in the circular and rectangular view. The tree is constructed by BIONJ based on genomic distance matrixes, and mid-point rooted. Branch lengths are logarithmically scaled from the root of the entire proteomic tree. The number inside the circular view represent the log scaled branch lengths based on the SG values (normalized tBLASTx score). MSK phage used in this study highlighted with red star for Rtp closely related phage is highlighted with blue star in rectangular view.

2 Supplementary Tables

2.1 Supplementary Table 1-A

Gene locus	Pfam number	Pfam ID	Pfam description
MSK_00001	1	PF01499	Herpesvirus UL25 family
MSK_000049	2	PF10153; PF00189	Uncharacterised conserved protein (DUF2361); Ribosomal protein S3, C-terminal domain
MSK_000015	1	PF00854	POT family
MSK_000044	2	PF02416; PF10439	mttA/Hcf106 family; Bacteriocin class II with double-glycine leader peptide
MSK_000063	1	PF06560:	Glucose-6-phosphate isomerase (GPI)
MSK_000011	1	PF00193:	Extracellular link domain
MSK_000033	1	PF08774	VRR-NUC domain
MSK_000030	1	PF08273	Zinc-binding domain of primase-helicase
MSK_000022	1	PF01555	DNA methylase
MSK_000040	3	PF02786; PF00196; PF12063	Carbamoyl-phosphate synthase L chain, ATP binding domain; Bacterial regulatory proteins, luxR family; Domain of unknown function (DUF3543)
MSK_000025	1	PF03320	Bacterial fructose-1,6-bisphosphatase, glpX-encoded
MSK_000031	1	PF08281	Sigma-70, region 4
MSK_000037	1	PF01565	FAD binding domain
MSK_000045	1	PF00959	Phage lysozyme
MSK_000072	2	PF04851; PF00270:	Type III restriction enzyme, res subunit; DEAD/DEAH box helicase

MSK_000028	5	PF12326; PF12090; PF02129; PF03244; PF01080	N-glycosylation protein; Spt20 family; X-Pro dipeptidyl-peptidase (S15 family); Photosystem I reaction centre subunit VI; Presenilin

Supplementary Table 1-B

Genes locus	Clan	number	Description
MSK_000028	CL0028.21	1	Alpha/Beta hydrolase fold
MSK_000040	CL0179.13	1	ATP-grasp superfamily
MSK_000011	CL0056.11	1	C-type lectin-like superfamily
MSK_000063	CL0029.19	1	Cupin fold
MSK_000037	CL0077.11	1	PCMH-like FAD binding
MSK_000044	CL0400.3	1	Double-Glycine leader-peptide cleavage motif
MSK_000040;MSK_000031	CL0123.17	2	Helix-turn-helix clan
MSK_000045	CL0037.13	1	Lysozyme-like superfamily
MSK_000015	CL0015.19	1	Major Facilitator Superfamily
MSK_000022	CL0063.24	1	FAD/NAD(P)-binding Rossmann fold Superfamily
MSK_000072; MSK_000072	CL0023.33	2	P-loop containing nucleoside triphosphate hydrolase superfamily
MSK_000033	CL0236.16	1	PD-(D/E)XK nuclease superfamily
MSK_000028	CL0130.10	1	Peptidase clan AD
MSK_000025	CL0171.10	1	inositol polyphosphate 1 phosphatase

			like superfamily
MSK_000030	CL0167.14	1	Zinc beta-ribbon

Supplementary Table 1-A: Gene Pfam table, showing the gene locu, Pfam number, Pfam ID and Pfam description. **1-B:** Pfam Clan table showing the gene locus, clan, number and description.

2.2 Supplementary Table 2

Locus ID	Organism	Identity	E-value	Protein ID	Swiss prot gene	Subject ID
MSK_000016	Escherichia phage N15	45.2	4.10E-48	Tail tip protein L	Gene 18	O64332
MSK_000017	Escherichia phage N15	45.8	8.50E-54	Tail tip assembly protein K	Gene 19	O64333
MSK_000018	Escherichia phage N15	43.5	5.30E-32	Tail tip assembly protein I	Gene20	O64334
MSK_000044	Escherichia phage T1	57.7	1.40E-17	Putative holin	13	Q6XQ99
MSK_000045	Escherichia phage T1	46.2	7.20E-30	Endolysin	12	Q6XQ98
MSK_000046	Escherichia phage T1	40	2.30E-15	U-spanin	11	Q6XQ97

Supplementary Table 2: Swiss-pro annotation table: annotation result achieved through swiss-pro database.

2.3 Supplementary Table 3

**Locus_tag**	**Nucleotide**	**strand**	**protein length**	**related protein**	**Blast p**				**Description related protein**
					score	E vale	% ID	Overlp (aa)	
MSK_00001	151-1236	+	361	QLF80554.1	700	0.0	96.96%	351	phage capsid and scaffold [Escherichia phage vB_EcoS_FP]
MSK_00002	1248-1466	+	72	YP_006987837.1	135	8e-39	95.83%	69	Hypothetical protein ACG-M12_0019
MSK_00003	1512-1757	+	81	YP_009900844.1	154	6e-46	93.83%	76	hypothetical protein OKDFICHP_00024
MSK_00004	1869-2813	+	314	YP_398971.1	627	0.0	96.18%	302	hypothetical protein rtp27 [Escherichia virus Rtp]
MSK_00005	2907-3152	+	81	YP_398972.1	160	1e-49	98.77%	80	hypothetical protein rtp28 [Escherichia virus Rtp]
MSK_00006	3194-3595	+	133	ACZ74599.1	262	4e-88	96.24%	128	hypothetical protein -Halo29 [Escherichia phage RES-2009a]
MSK_00007	3592-3963	+	123	YP_398974.1	231	3e-76	93.50%	115	conserved phage-related protein [Escherichia virus Rtp]
MSK_00008	3956-4405	+	149	YP_398975.1	197	5e-62	66.43%	93	conserved phage-related protein [Escherichia virus Rtp]
MSK_00009	4395-4796	+	66	YP_398976.1	131	1e-37	95.45%	63	hypothetical protein hypothetical protein

									rtp32 [Escherichia virus Rtp]
MSK_000010	4812-5468	+	218	YP_398977.1	416	2e-146	93.12%	203	putative major tail protein [Escherichia virus Rtp]
MSK_000011	5578-5826	+	82	YP_009789213.1	161	8e-50	98.78%	81	hypothetical protein [Escherichia phage vB_EcoS-IME253]
MSK_000012	5866-6180	+	104	YP_009789325.1	207	3e-67	99.04%	103	tail assembly chaperone [Escherichia phage vB_EcoS_CEB_EC3a]
				YP_398979.1	198	6e-64	95.19%	102	hypothetical protein rtp35 [Escherichia virus Rtp]
MSK_000013	6189-6500	+	103	YP_398980.1	205	1e-66	96.12%	99	hypothetical protein rtp36 [Escherichia virus Rtp]
MSK_000014	6536-9514	+	992	YP_006987848.1	1826	0.0	96.17%	954	putative tail tape measure protein [Ecoli phage vB_EcoS_ACG-M12]
MSK_000015	9545-9895	+	116	YP_398982.1	218	2e-71	88.79%	103	phage minor tail protein [Escherichia virus Rtp]
MSK_000016	9935-10690	+	251	YP_398983.1	503	6e-180	96.41%	242	phage minor tail protein [Escherichia virus Rtp]
MSK_00001	10701-	+	252	YP_398985.1	503	2e-179	94.44%	238	phage tail tip, assembly protein

7	11459								[Escherichia virus Rtp]
MSK_000018	11440-12012	+	190	YP_398986.1	367	7e-128	94.21%	179	phage tail tip, assembly protein [Escherichia virus Rtp]
MSK_000019	12093-15479	+	1128	YP_398987.1	2216	0.0	95.92%	1082	putative tail fiber protein [Escherichia virus Rtp] rtp 43
MSK_000020	16473-15511	-	320	QHJ72654.1	626	0.0	96.56%	309	hypothetical protein [Escherichia phage 2725-N35]
MSK_000021	16787-16473	-	104	YP_398989.1	118	2e-32	73.75%	59	putative phage lipoprotein [Escherichia virus Rtp] rtp 45
				YP_009036027.1	166	3e-51	87.78%	79	hypothetical protein [Escherichia phage e4/1c]
MSK_000022	17473-16781	-	230	AXC39488.1	293	4e-97	61.11%	143	DNA adenine methyltransferase YhdJ
MSK_000023	17579-17845	+	88	None					
MSK_000024	18015-18209	+	64	YP_398990.1	111	1e-29	94.44%	51	hypothetical protein rtp46 [Escherichia virus Rtp]
MSK_000025	18213-19178	+	321	YP_398991.1	587	0.0	92.21%	296	phage exonuclease VIII (RecE) [Escherichia virus Rtp]
MSK_00002	19218-	+	61	None					

6	19403								
MSK_00002 7	19403-20053	+	216	YP_398992.1	435	6e-154	97.69%	211	phage associated recombinase [Escherichia virus Rtp]
MSK_00002 8	20095-20529	+	144	YP_009795741.1	226	6e-74	85.42%	123	hypothetical protein [Escherichia phage DTL]
				YP_398993.1	226	1e-73	86.11%	124	putative single-stranded DNA binding protein [Escherichia virus Rtp]
MSK_00002 9	23015-20565	-	816	YP_398994.1	209	2e-52	73.61%	106	phage tail fibers [Escherichia virus Rtp] rtp 50
				YP_009018638.1	397	4e-120	67.91%	218	gp24 [Escherichia phage phiEB49]
MSK_00003 0	24030-23104	-	308	YP_398995.1	358	2e-120	56.03%	172	DNA primase [Escherichia virus Rtp]
				QBQ80880.1	576	0.0	86.69%	267	putative primase-helicase [Escherichia phage vB_EcoS_MM01]
MSK_00003 1	24578-24096	-	160	YP_009789237.1	320	2e-110	97.45%	153	hypothetical protein [Escherichia phage vB_EcoS-IME253]
				QLF84981.1	314	4e-108	96.18%	151	transcriptional regulator [Escherichia phage vB_EcoD_SU57]664
MSK_00003 2	24680-26674	+	664	UniProtKB:P33919					Putative DNA repair helicase RadD

				YP_398997.1	1121	0.0	81.82%	540	putative ATP-dependent helicase [Escherichia virus Rtp]
MSK_000033	26671-27090	+	139	QHJ72642.1	270	5e-91	93.53%	130	hypothetical protein [Escherichia phage 2725-N35]
MSK_000034	27329-27571	+	80	YP_009795749.1	119	2e-33	89.06%	57	hypothetical protein [Escherichia phage DTL]
				YP_398999.1	97.8	7e-25	78.79%	52	hypothetical protein rtp55 [Escherichia virus Rtp]
MSK_000035	27571-27789	+	72	YP_009795750.1	107	5e-28	72.22%	52	hypothetical protein [Escherichia phage DTL]
				YP_399000.1	104	1e-27	68.06%	49	hypothetical protein rtp56 [Escherichia virus Rtp]
MSK_000036	27792-27917	+	41	YP_399002.1	65.5	5e-13	70.73%	29	hypothetical protein rtp58 [Escherichia virus Rtp]
MSK_000037	27914-28162	+	82	YP_399003.1	160	3e-49	93.90%	77	hypothetical protein rtp59 [Escherichia virus Rtp]
MSK_000038	28165-28311	+	48	WP_167808460.1	42.4	0.012	54.29%	19	hypothetical protein [Yokenella regensburgei]
MSK_000039	28314-28565	+	83	YP_399004.1	164	7e-51	93.90%	77	hypothetical protein rtp60 [Escherichia virus Rtp]

MSK_000040	28647-29777	+	376	QLF85013.1	748	0.0	95.21%	358	hypothetical protein [Escherichia phage vB_EcoD_SU57]
				YP_399005.1	563	0.0	69.95%	263	hypothetical protein rtp61 [Escherichia virus Rtp]
MSK_000041	29852-30325	+	157	YP_006987881.1	306	8e-105	92.36%	145	polynucleotide kinase/phosphatase<br><br>[Escherichia phage vB_EcoS_ACG-M12]
MSK_000042	30328-30879	+	183	QLF84977.1	356	0.0	96.23%	173	ATPase [Escherichia phage vB_EcoD_SU57]
MSK_000043	30951-31127	+	58	QLF80606.1	122	3e-35	100.00%	58	hypothetical protein FP_0059 [Escherichia phage vB_EcoS_FP]
				YP_399006.1	93.6	1e-23	79.63%	43	hypothetical protein rtp62 [Escherichia virus Rtp]
MSK_000044	31258-31473	+	71	YP_009795695.1	137	1e-40	98.59%	70	putative holing [Escherichia phage DTL]
				YP_399007.1	91.7	1e-22	95.77%	68	putative holin [Escherichia virus Rtp]
MSK_000045	31474-31959	+	161	UniProtKB:P78285					Lysozyme RrrD
				YP_009789354.1	310	2e-106	93.17%	150	endolysin [Escherichia phage vB_EcoS_CEB_EC3a]

				YP_399008.1	282	3e-95	89.44%	144	putative endolysin [Escherichia virus Rtp]
MSK_000046	31935-32324	+	129	VUF54959.1	112	4e-29	47.93%	58	phage lipoprotein [Escherichia phage Stevie_ev116]
				YP_009789355.1	190	6e-60	93.75%	120	putative membrane protein<br><br>[Escherichia phage vB_EcoS_CEB_EC3a]
				YP_399009.1	217	8e-71	91.60%	109	hypothetical protein r**tp45**[Escherichia virus Rtp]
MSK_000047	32566-32357	-	69	YP_009177394.1	84.3	9e-20	62.69%	42	hypothetical protein phiTE_096 [Pectobacterium phage phiTE]
MSK_000048	33012-32677	-	111	YP_009795698.1	214	5e-70	93.64%	103	hypothetical protein [Escherichia phage DTL]
				YP_399010.1	172	1e-53	90.00%	91	hypothetical protein rtp66 [Escherichia virus Rtp]
MSK_000049	34685-33102	-	527	YP_399011.1	1057	0.0	96.39%	508	hypothetical protein rtp67 [Escherichia virus Rtp]
MSK_000050	34875-34717	-	52	QLF85009.1	72	2e-15	94.74%	36	hypothetical protein [Escherichia phage vB_EcoD_SU57]
MSK_00005	35111-	-	79	YP_399012.1	145	8e-44	88.61%	70	hypothetical protein rtp68 [Escherichia

1	34872								virus Rtp]
MSK_00005 2	35461-35108	-	117	YP_399013.1	197	5e-63	80.34%	94	hypothetical protein rtp69 [Escherichia virus Rtp]
MSK_00005 3	35622-35458	-	54	YP_399014.1	95.9	1e-24	88.24%	45	hypothetical protein rtp70 [Escherichia virus Rtp]
MSK_00005 4	35801-35622	-	59	QLF85006.1	88.2	2e-21	81.03%	47	hypothetical protein [Escherichia phage vB_EcoD_SU57]
MSK_00005 5	36017-35856	-	53	YP_399016.1	111	6e-31	98.11%	52	hypothetical protein rtp72 [Escherichia virus Rtp]
MSK_00005 6	36211-36029	-	60	QLF80600.1	111	1e-30	90.00%	54	hypothetical protein FP_0066 [Escherichia phage vB_EcoS_FP]
MSK_00005 7	36450-36211	-	79	YP_399017.1	154	2e-47	92.41%	73	hypothetical protein rtp73 [Escherichia virus Rtp]
MSK_00005 8	36640-36461	-	59	YP_399018.1	99.4	5e-26	89.09%	49	hypothetical protein rtp74 [Escherichia virus Rtp]
MSK_00005 9	37343-37864	+	173	QLF80566.1	313	5e-107	93.64%	162	hypothetical protein FP_0001 [Escherichia phage vB_EcoS_FP]
				YP_398945.1	197	2e-61	56.98%	98	hypothetical protein rtp1[Escherichia virus Rtp]
MSK_00006	37864-	+	38	QLF80565.1	84.7	4e-19	100.00	38	hypothetical protein FP_0002

0	37980						%		[Escherichia phage vB_EcoS_FP]
MSK_00006 1	38011-38283	+	76	YP_277486.1	98.6	7e-24	64.47%	49	hypothetical protein JK_46 [Escherichia virus KP26]
MSK_00006 2	38412-38627	+	71	YP_009789913.1	131	3e-38	88.73%	63	hypothetical protein ESCO41_00029<br><br>[Escherichia phage vB_EcoS_ESCO41]
MSK_00006 3	38644-38886	+	80	QLF85042.1	49.7	7e-06	49.12%	28	hypothetical protein [Escherichia phage vB_EcoD_SU57]
MSK_00006 4	38888-39079	+	63	YP_006987823.1	50.8	1e-06	50.00%	22	hypothetical protein ACG-M12_0005<br><br>[Escherichia phage vB_EcoS_ACG-M12]
MSK_00006 5	39081-39299	+	72	QLF80593.1	138	4e-41	93.06%	67	hypothetical protein FP_0003 [Escherichia phage vB_EcoS_FP]
				YP_398951.1	97.4	9e-25	70.13%	54	hypothetical protein rtp7 [Escherichia virus Rtp]
MSK_00006 6	39526-39669	+	47	YP_009900856.1	94.7	2e-24	95.74%	45	hypothetical protein OKDFICHP_00037<br><br>[Escherichia phage vB_EcoS-12210I]
				QLF80610.1	56.2	3e-09	69.23%	27	hypothetical protein FP_0004 [Escherichia phage vB_EcoS_FP]

				YP_398953.1	55.5	7e-09	68.42%	26	hypothetical protein rtp9 [Escherichia virus Rtp]
MSK_000067	39744-39914	+	56	YP_009789925.1	60.1	2e-10	77.19%	44	hypothetical protein ESCO41_00041<br><br>[Escherichia phage vB_EcoS_ESCO41]
MSK_000068	39916-40155	+	79	ATI17066.1	134	2e-39	79.75%	63	hypothetical protein [Escherichia phage IMM-001]
				QLF80589.1	125	7e-36	72.15%	57	hypothetical protein FP_00011 [Escherichia phage vB_EcoS_FP]
				YP_398959.1	121	3e-34	72.15%	57	hypothetical protein rtp7 [Escherichia virus Rtp]
MSK_000069	40479-40556	+							tRNA-Arg(tct)
MSK_000070	40584-40769	+	61	YP_398960.1	117	4e-33	90.16%	61	hypothetical protein rtp16 [Escherichia virus Rtp]
MSK_000071	41065-41571	+	168	QHJ72678.1	338	4e-117	98.21%	165	terminase, small subunit [Escherichia phage 2725-N35]
				QLF80568.1	336	3e-116	97.02%	163	terminase, small subunit [Escherichia phage vB_EcoS_FP]
				YP_398963.1	329	1e-113	94.64%	159	putative terminase small subunit [Escherichia virus Rtp]

MSK_00007 2	41590-43155	+	521	YP_009789200.1	1053	0.0	97.12%	506	terminase large subunit [Escherichia phage vB_EcoS-IME253]
				YP_398965.1	913	0.0.	82.47%	428	putative terminase large subunit [Escherichia virus Rtp]
MSK_00007 3	43204-44472	+	422	YP_009789202.1	828	0.0	95.26%	402	portal protein [Escherichia phage vB_EcoS-IME253]
				YP_398967.1	827	0.0	95.26%	402	putative portal protein [Escherichia virus Rtp]
				QLF80552.1	806	0.0	94.24%	393	portal protein [Escherichia phage vB_EcoS_FP]

Supplementary Table 3: Annotation of the genome MSK bacteriophage, locus tags, ORFs start and end point, protein length, number of related proteins accession no and their description and BLASTp hit homology. Among these 73 predicted ORFs, 47 ORF shows homology with Rtp bacteriophage
