# Supplementary material for: Virulent Drexlervirial Bacteriophage MSK, Morphological and Genome Resemblance With Rtp Bacteriophage Inhibits the Multidrug-Resistant Bacteria
Source: Front Microbiol. 2021 Aug 24;12:706700. doi: 10.3389/fmicb.2021.706700 (PMC8421802; doi:10.3389/fmicb.2021.706700)

PHACTS (index.php)

Home (index.php) Upload (upload.php) Retrieve (retrieve.php) FAQ (faq.php)

Contact (https://groups.google.com/d/forum/edwardslabtools)

Results Page

Log for job RID: 16219685382

```
11:50:13 Uploaded file: MSK.faa
11:50:14 Starting gram Analysis
11:50:14 Starting lifestyle Analysis
11:50:15 Your job 8404531 (s_gram.sh) has been submitted
11:50:15 Your job 8404532 (s_lifestyle.sh) has been submitted
11:53:15 Completed gram Analysis
11:53:15 Completed lifestyle Analysis
```

Lifestyle:

Analysis Statistics

Ten iterations of PHACTS were performed using the default settings. The phage was **non-confidently** predicted as having a **Lytic lifestyle**.

Predicted Class	Averaged Probability	Standard Deviation
Lytic	0.513	0.046

Probability that phage is **Lytic**

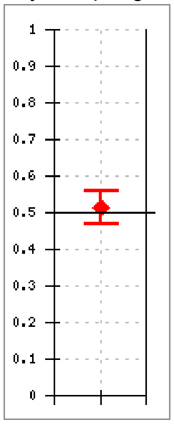

Gram-stain of host:

Analysis Statistics

Ten iterations of PHACTS were performed using the default settings. The phage was **non-confidently** predicted as infecting a **Gram Negative** host.

Predicted Class	Averaged Probability	Standard Deviation
Negative	0.53	0.036

Probability that phage is **Negative**

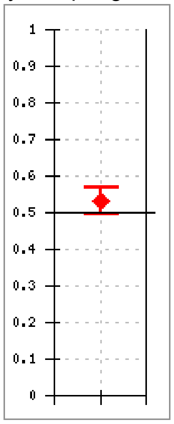

Supplement: Supplementary file 3 [file Data_Sheet_3.PDF]
